# Binary vector copy number engineering improves *Agrobacterium*-mediated transformation

**DOI:** 10.1038/s41587-024-02462-2

**Published:** 2024-11-04

**Authors:** Matthew J. Szarzanowicz, Lucas M. Waldburger, Michael Busche, Gina M. Geiselman, Liam D. Kirkpatrick, Alexander J. Kehl, Claudine Tahmin, Rita C. Kuo, Joshua McCauley, Hamreet Pannu, Ruoming Cui, Shuying Liu, Nathan J. Hillson, Jacob O. Brunkard, Jay D. Keasling, John M. Gladden, Mitchell G. Thompson, Patrick M. Shih

**Affiliations:** 1 Joint BioEnergy Institute, Emeryville, CA, USA.; 2 Environmental Genomics and Systems Biology Division, Lawrence Berkeley National Laboratory, Berkeley, California, USA.; 3 Department of Plant and Microbial Biology, University of California, Berkeley, Berkeley, CA, USA.; 4 Department of Bioengineering, University of California, Berkeley, Berkeley, CA, USA.; 5 Biological Systems and Engineering Division, Lawrence Berkeley National Laboratory, Berkeley, CA, USA.; 6 Laboratory of Genetics, University of Wisconsin-Madison, Madison, WI, USA.; 7 Sandia National Laboratories, Livermore, CA, USA.; 8 Department of Chemistry, University of California, Davis, Davis, CA, USA.; 9 Department of Chemical and Biomolecular Engineering, University of California, Berkeley, Berkeley, CA, USA.; 10 QB3, University of California, Berkeley, Berkeley, CA, USA.; 11 Center for Biosustainability, Danish Technical University, Kongens Lyngby, Denmark.; 12 Innovative Genomics Institute, Berkeley, CA, USA.

## Abstract

The copy number of a plasmid is linked to its functionality, yet there have been few attempts to optimize higher-copy-number mutants for use across diverse origins of replication in different hosts. We use a high-throughput growth-coupled selection assay and a directed evolution approach to rapidly identify origin of replication mutations that influence copy number and screen for mutants that improve *Agrobacterium*-mediated transformation (AMT) efficiency. By introducing these mutations into binary vectors within the plasmid backbone used for AMT, we observe improved transient transformation of *Nicotiana benthamiana* in four diverse tested origins (pVS1, RK2, pSa and BBR1). For the best-performing origin, pVS1, we isolate higher-copy-number variants that increase stable transformation efficiencies by 60–100% in *Arabidopsis thaliana* and 390% in the oleaginous yeast *Rhodosporidium toruloides*. Our work provides an easily deployable framework to generate plasmid copy number variants that will enable greater precision in prokaryotic genetic engineering, in addition to improving AMT efficiency.

*Agrobacterium*-mediated transformation (AMT) is an indispensable tool in both plant and fungal biotechnology for transgene insertion into target cells^1–3^. By replacing the native oncogenic genes used by the bacterial pathogen *Agrobacterium tumefaciens* with user-defined DNA sequences, the innate DNA transfer capacity of this bacterium can be exploited for untargeted DNA insertions into diverse plant, fungal and mammalian cell lines^4–7^. Initial improvements to AMT involved relocating two DNA sequences known as the left and right border (LB and RB) from the native ~200-kb tumor-inducing (Ti) plasmid to a smaller helper plasmid known as a binary vector^8^. Any DNA contained between the LB and RB is mobilized as a transfer DNA (T-DNA) and inserted into target cells with help from virulence (*vir*) genes contained within the Ti plasmid, enabling tractable engineering by cloning different sequences into the T-DNA region^1^. The simplicity of changing the transgene target by customizing the sequence between the LB and RB has made AMT an important tool for agricultural biotechnology, bioenergy crop engineering and synthetic biology.

Despite the genetic revolution that AMT initiated, efficient transformation is still a considerable bottleneck for genetic engineering in most plant species. Decades of optimization of AMT have identified induction conditions^9,10^, strains of *A. tumefaciens*^4^ and enhancements to *vir* gene expression that have increased AMT efficiency in numerous plant species^11^. In addition to the transgene-harboring binary vector, some protocols have used a second introduced plasmid, termed a ternary vector, which overexpresses various genes involved in *Agrobacterium* virulence to improve AMT efficiencies in recalcitrant plants such as maize^11^. Such work has inspired more recent research to completely refactor the Ti plasmid that harbors the *vir* genes^12^, laying the groundwork for fine-tuned engineering of individual virulence components to further enhance AMT. Even with these improvements, transformation efficiencies remain low for most genetic backgrounds and limit the scale and throughput of genetic engineering projects, presenting a need for improved tools to better control and enhance the AMT process.

While many improvements to AMT have focused on either altering *vir* gene expression or sequences within the T-DNA^1,2,13,14^, relatively little work has been conducted to engineer the binary vector backbone itself. Binary vectors consist of a T-DNA region, a bacterial selectable marker and an origin of replication (ORI) to enable proper replication and maintenance of the plasmid in *Agrobacterium*. Within plasmid terminology, the term ORI refers to both the origin of vegetative replication (oriV, as cataloged by Dong et al.^15^) where plasmid replication initiates and the coding sequences for specific proteins that bind to the oriV or host replication factors to regulate plasmid copy number, stability and partitioning such as Rep, Stb and Par proteins, respectively. In this manuscript, the term ORI refers to the entire DNA sequence that mediates plasmid replication, which includes the oriV and *trans*-acting factors such as Rep proteins^16^. For binary vectors, the ORI, which dictates both host range and copy number, is known to impact AMT efficiency in plants^16–19^. Zhi et al. postulated a direct relationship between binary vector copy number and maize transformation efficiency through a comparison of three binary vector ORIs, suggesting that higher copy numbers could be used to improve AMT^17^. A similar comparison of four ORIs conducted by Oltmanns et al. in *Arabidopsis thaliana* and a different cultivar of maize uncovered a more complicated relationship that implicated the strain of *A. tumefaciens*, the ORI and the plant genetic background as factors that independently influence transformation efficiencies^18^. Notably, however, neither study evaluated the impact of copy number variants within the same ORI, making it difficult to isolate the impact of copy number alone while controlling for intrinsic differences inherent to each ORI. While there is evidence that different ORI copy numbers influence AMT efficiency, there has never been an attempt to systematically modify this variable for the binary vector. The best attempt to date at direct manipulation of the binary vector ORI to alter AMT outcomes was by Vaghchhipawala et al., who evaluated a single higher-copy-number mutant of the pRi ORI and found no improvement in stable transformation^19^. Thus, a more comprehensive screen of ORI variants is needed to better understand the relationship among ORI identity, plasmid copy number and AMT transformation outcomes.

In prokaryotic synthetic biology, it has long been known that the copy number of a plasmid can dramatically impact engineered metabolic pathways and the functionality of synthetic circuits^20–24^. As plasmid copy number influences transgene expression magnitude and metabolic load, previous work has been conducted to engineer plasmid copy number, particularly in narrow-host-range ORIs commonly used in *Escherichia coli* such as pMB1 and pSC101 derivatives^21,25,26^. For broad-host-range ORIs that are used within *A. tumefaciens*, engineering efforts have been extremely limited with only a few mutants isolated^20,27–30^. To evaluate the impact of plasmid copy number across multiple broad-host-range ORIs used for AMT, a high-throughput screen is needed to identify and characterize numerous copy number variants. To date, however, no generally applicable method exists that can be applied to diverse ORIs to systematically screen for copy number diversity.

To specifically evaluate the impact of binary vector copy on AMT, we sought to generate copy number variants in four broad-host-range origins (RK2, pVS1, pSa and BBR1) that have been widely used in *Agrobacterium* and prokaryotic biotechnology more broadly. To accomplish this, we leveraged a high-throughput growth-coupled selection assay to rapidly identify ORI mutations that influence copy number. Across all four origins, we were able to use a transient expression assay in *Nicotiana benthamiana* to rapidly screen for mutants that could improve AMT efficiency. Using top candidate plasmid variants from this screen, we were able to notably improve stable transformation in both *A. thaliana* and the oleaginous yeast *Rhodosporidium toruloides*. Thus, our work demonstrates the impact of binary vector backbone engineering on AMT across kingdoms, creating an easily deployable strategy to improve transformation.

## Results

### Directed evolution pipeline to diversify plasmid copy number

As previous work suggested a relationship between binary vector copy number and AMT transformation efficiency^17^, we sought to develop a method to systematically select for higher-copy-number mutants across diverse ORIs. Despite numerous intrinsic differences, many ORIs use analogous but nonhomologous replication initiation proteins to control plasmid replication and copy number. These nonhomologous RepA proteins share a general function of binding in *cis* to a motif within the oriV of the plasmid ORI to recruit various endogenous bacterial factors to enable plasmid replication^31^. Previous studies demonstrated that mutations impacting RepA proteins can alter plasmid copy number for diverse ORIs including pSC101 (ref. 21), BBR1 (ref. 20), RK2 (refs. 29,30) and pVS1 (ref. 32). To leverage this general property, the entire *repA* open reading frame (ORF) was randomly mutagenized using error-prone PCR (epPCR) for the pVS1, pSa, RK2 and BBR1 ORIs. These ORIs are derived from unique incompatibility groups and their respective RepA proteins share no sequence homology (Supplementary Fig. 1). For each ORI, these mutagenized ORFs were then built into a selection vector and ~100,000 colonies were pooled per ORI to create four mutant libraries within the C58C1 strain of *A. tumefaciens*.

**Fig. 1 |:**
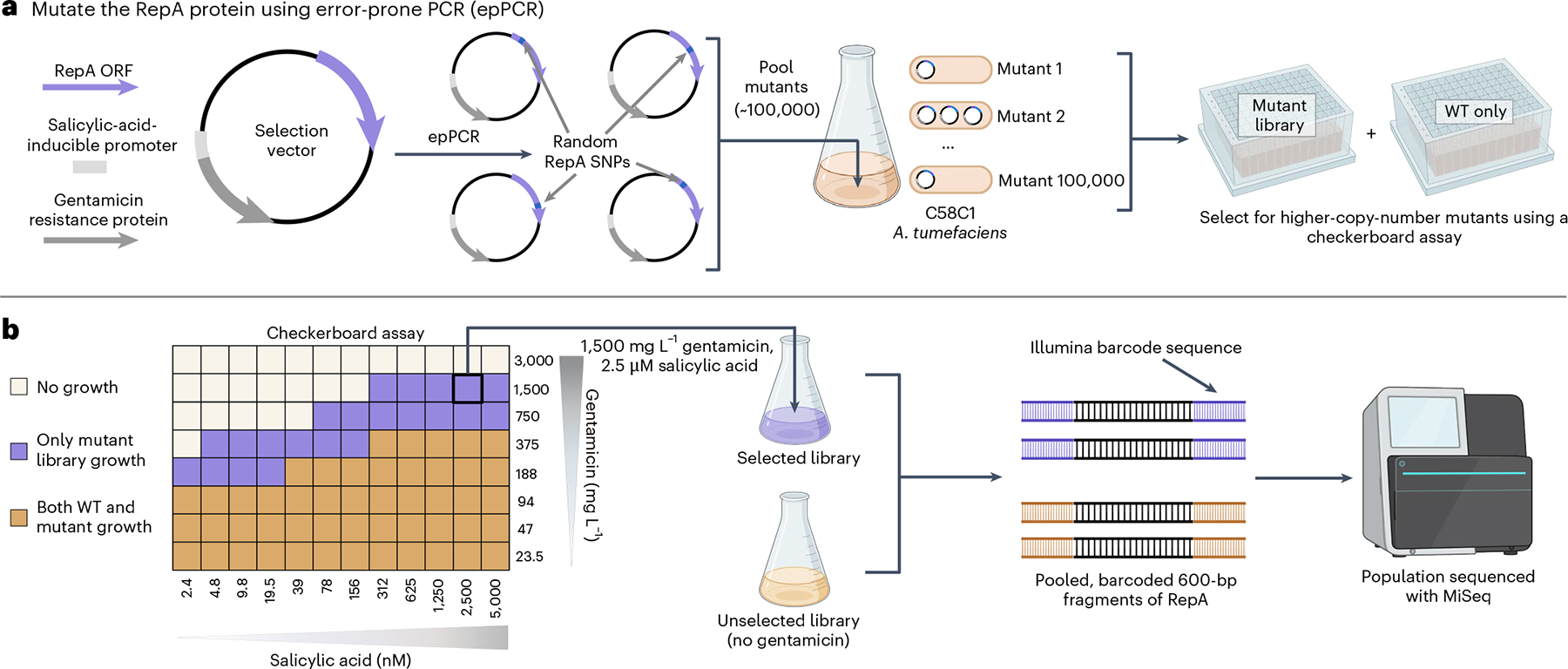
Directed evolution pipeline to generate plasmid copy number variants using next-generation sequencing. **a**, Schematic of the *repA* mutagenesis method for the four ORIs. A selection vector was designed with a gentamicin resistance gene driven by a salicylic-acid-inducible promoter to enable selection in a checkerboard assay. The *repA* ORF was mutagenized with epPCR and ~100,000 mutants per ORI were pooled to create a mutant library. **b**, Example checkerboard data from pVS1 (other ORI data in Supplementary Fig. 2) along with a depiction of the population sequencing strategy. Selection conditions within the checkerboard assay that were only permissible for growth of the mutant library are shown in purple; conditions that permitted growth for both the WT and the mutant populations are shown in brown.

To select for higher-copy-number variants within these libraries, we use a directed evolution assay that coupled plasmid copy number to bacterial antibiotic tolerance (Fig. 1). This survival-coupled selection identified wild type (WT)-lethal conditions that were permissible for growth of the mutant population, enriching higher-copy-number mutants within this population (Supplementary Fig. 2). For each selected library, the entire population’s *repA* ORFs were sequenced using an Illumina MiSeq alongside unselected library controls to evaluate the selective pressure of each RepA residue on survival.

**Fig. 2 |:**
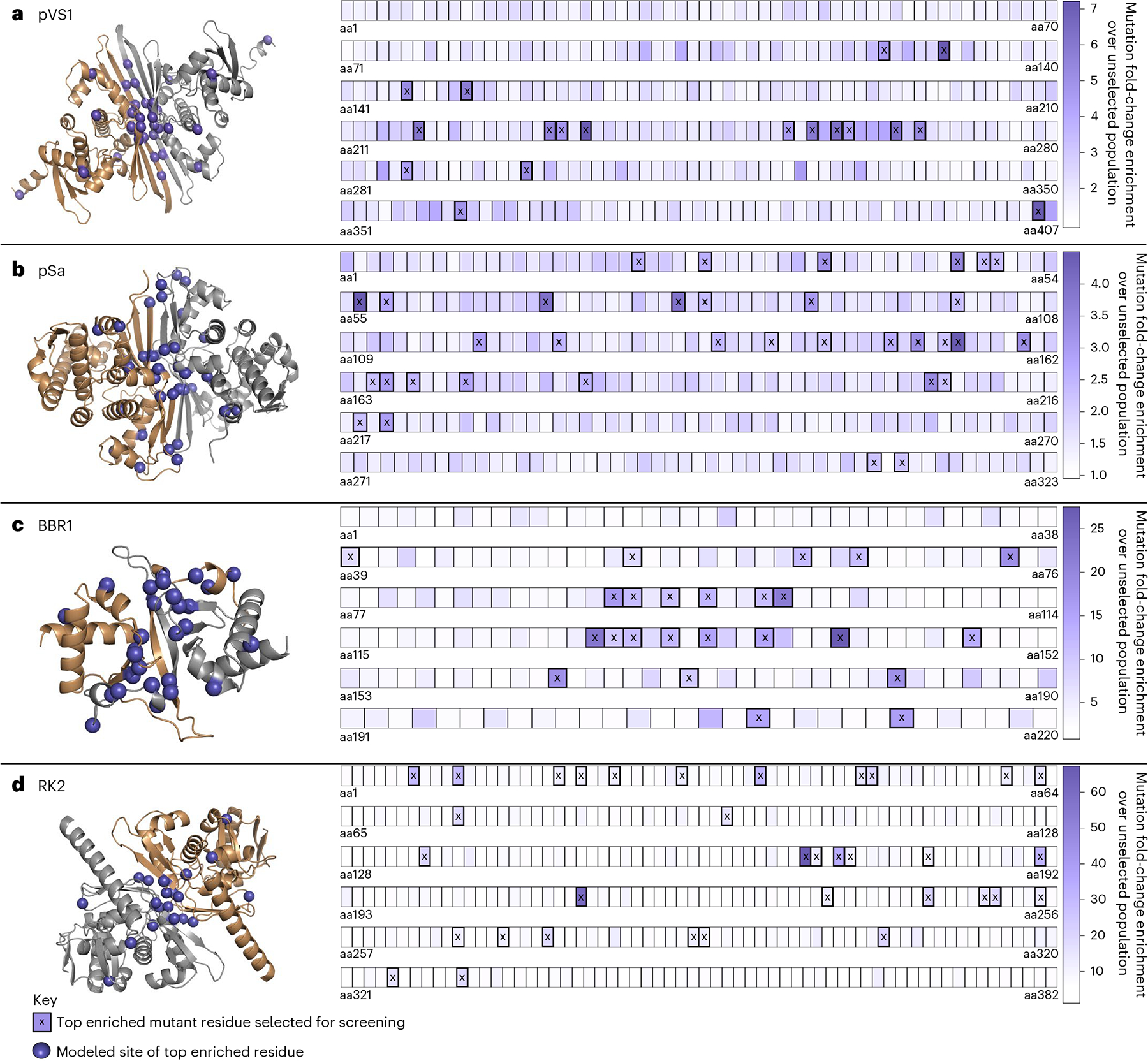
Enriched residue sites after copy number variant selection. Left, AlphaFold structures of the RepA proteins from each of the four ORIs screened: pVS1 (**a**), pSa (**b**), BBR1 (**c**) and RK2 (**d**). Right, primary structure of each protein with each box corresponding to a single residue. The shading of these boxes corresponds to the fold-change enrichment of a mutation impacting this residue within the selected population compared to the unselected control. Any residue that had an enrichment above a cutoff threshold for yielding the top ~20 residues is marked with an ‘X’, representing the sites with the strongest selective pressure. These residues are depicted as a purple orb on the AlphaFold models. These models focus on the structured dimerization interface, and unstructured N-terminal and C-terminal tails were trimmed for compaction. These tails, along with a quality of model analysis, are depicted in Supplementary Fig. 5.

This whole-population sequencing approach enabled the quantification of mutant enrichment at every residue of diverse RepA proteins (Fig. 2 and Supplementary Fig. 3). By comparing mutant distribution frequencies between the unselected and selected population, residues that contributed to the survival of the mutants in WT-lethal conditions can be identified. For the RK2 and BBR1 ORIs, large fold-change enrichments for specific nucleotide positions within the selected population were found that reached 63.8-fold and 26.7-fold over the unselected population. The pVS1 and pSa ORIs had notably lower fold-change enrichments reaching 7.2-fold and 4.4-fold, respectively. Across the four ORIs, we observed >2-fold enrichment for 38, 118, 126 and 150 nucleotide positions for pSa, pVS1, BBR1 and RK2. Mutations that were significantly enriched in the selected population compared to the unselected control were putatively associated with a higher-copy-number phenotype (Supplementary Table 1).

Mapping selected residues onto a RepA AlphaFold-generated model showed a significant enrichment of mutation sites on the predicted dimerization interfaces for all four ORIs (Supplementary Fig. 4). Some RepA proteins are postulated to regulate plasmid copy number through a ‘handcuffing’ process in which monomeric RepA promotes plasmid replication while the dimerized form inhibits further polymerase activity, finalizing plasmid copy number^33^. It has been hypothesized that mutations that weaken the affinity of RepA to itself may reduce dimerization and enable additional replication, resulting in a higher final plasmid copy number^21^; the enrichment of selected residues on the dimerization interface for the four nonhomologous RepA proteins screened supports this notion. From this dataset, we chose ~20 residues per ORI that were found to be highly enriched for a mutation corresponding to an amino acid substitution to further characterize copy number and AMT performance in a *N. benthamiana* transient expression assay.

### ORI copy mutants enhance plant transient transformation

**Fig. 3 |:**
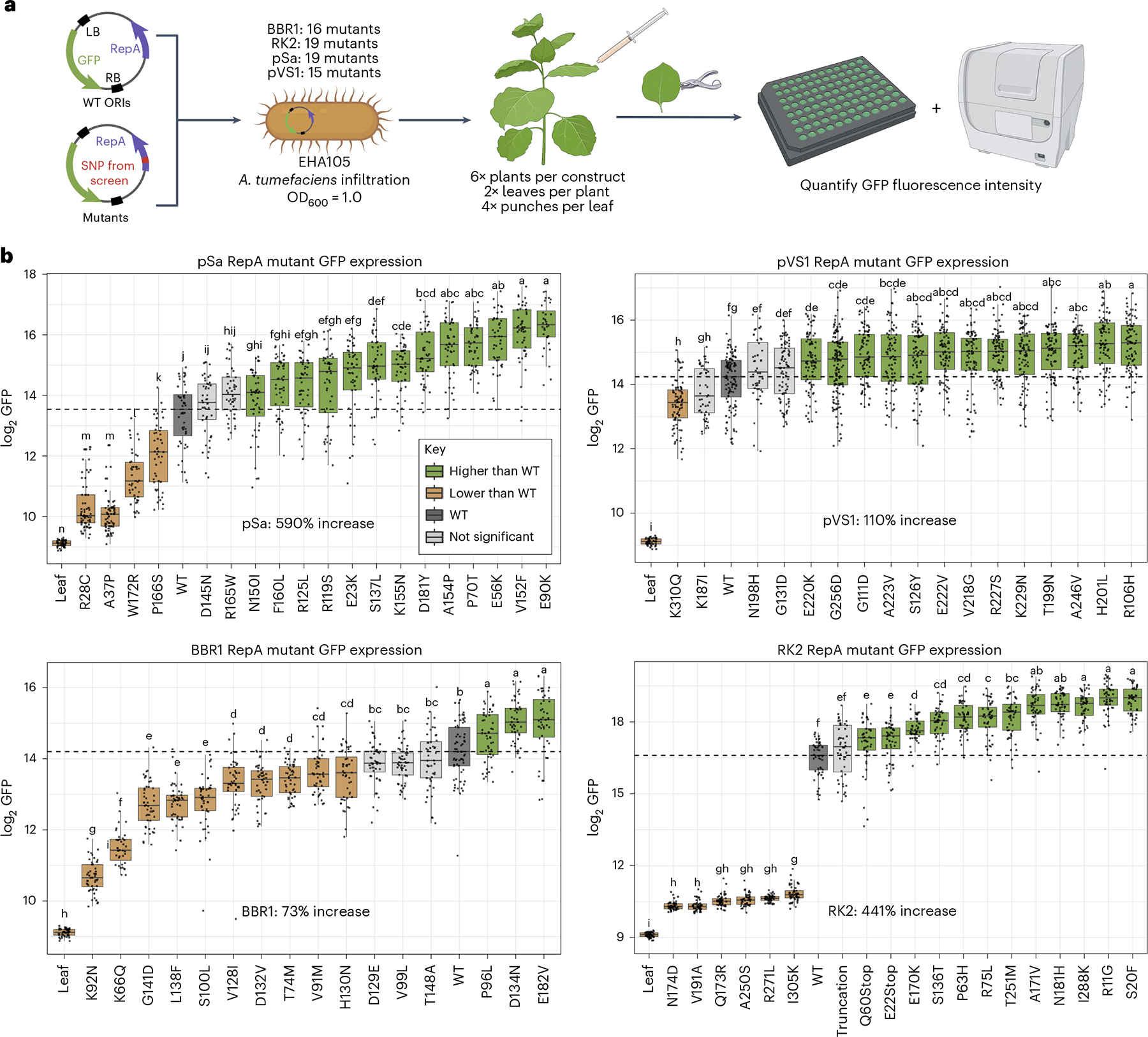
Screening RepA mutants for improved *N. benthamiana* transient transformation. **a**, Schematic of the transient expression assay in *N. benthamiana* used to screen the 71 mutants **b**, Box plots depicting the measured GFP output in *N. benthamiana* leaf discs for each construct (*n* = 48). Each point on the plot is the measured GFP fluorescence intensity of a single leaf disc. Boxes shaded in green were determined to have significantly higher GFP expression than the WT origin construct by a two-sided Tukey’s honestly significant difference (HSD) test (*P* < 0.05); light-gray boxes have nonsignificant differences while brown boxes have significantly lower expression levels. The dashed horizontal line in each plot corresponds to the median GFP output value for the WT of each ORI. Letters above each box plot correspond to the significance group as computed by Tukey’s HSD test. The upper and lower bounds of each box plot represent the 75th and 25th percentiles of the data, respectively, with the median value depicted as the center line; the whiskers extend up to 1.5× the interquartile range.

Residues that were found to be significantly enriched in our selection were cloned into uniform plant expression binary vectors. For a given ORI, identical binary vectors consisting of a constitutive plant promoter driving green fluorescent protein (GFP) were created, varying by one single-nucleotide polymorphism (SNP) within the *repA* ORF corresponding to one of the selected mutations. These vectors were transformed into the EHA105 strain of *A. tumefaciens* and used to screen the impact of single *repA* SNPs in a transient expression assay in *N. benthamiana* (Fig. 3a). Because the binary vectors for a given ORI were identical except for a single SNP in *repA*, differences in plant GFP output were attributed to the impact of this SNP on AMT efficiency. A total of 71 candidates across all origins were screened, and mutants for all four ORIs were identified that significantly increased GFP output relative to their WT forms (Fig. 3b and Supplementary Table 1).

We found the pSa ORI to have the largest fold-change increase in expression, with the E90K mutant having a 6.9-fold increase in GFP output relative to WT pSa. Furthermore, pSa had the greatest number of mutants that showed improvement over the WT form with 13 of the 19 mutants tested having significantly higher expression. This ORI also had the largest dynamic range of GFP expression, which spanned 28-fold over the 19 total mutants, significantly extending above and below WT output levels. These results are consistent with the known low WT copy number of this ORI in *Agrobacterium*, which potentially enables large improvements to be made by increasing the copy number^18^.

The RK2 origin also showed a substantial increase in plant GFP output, with the S20F mutant showing a 5.4-fold increase in expression. Notably for this ORI, six of the 19 mutants screened had extremely low GFP expression coupled with an observed slow growth phenotype, forming two distinct groups of high-expressing or low-expressing mutants rather than the gradient of outputs observed in other ORIs.

Of the four ORIs used in this study, pVS1 had the highest GFP output in its WT form (Supplementary Fig. 6). As RK2 was used in a pilot experiment and found to increase GFP expression 5.4-fold above WT levels for the best mutant, a lower-strength constitutive promoter, pCL2 (ref. 34), was used to screen pVS1 mutants to prevent saturating the machine’s GFP channel. A 2.1-fold increase in GFP output was observed for the best-performing pVS1 mutant, R106H. The BBR1 ORI was also found to have a high level of expression in its WT form; thus, the same pCL2 promoter was used to drive GFP expression in planta. For the 16 BBR1 mutants screened, ten had significantly lower GFP outputs than the WT form, making this the only ORI with a majority of mutants having decreased expression. The top-performing mutant, E182V, showed a 1.7-fold increase in GFP output relative to WT BBR1. As no previous work established the copy number of BBR1 in *A. tumefaciens*, this result suggests that increasing the copy number may not be as beneficial for this origin.

Collectively, our results demonstrate that manipulation of the binary vector ORI greatly expands the dynamic range of T-DNA delivery to target cells, creating an additional orthogonal variable to regulate transient expression that is independent of promoter and *Agrobacterium* strain selection. All ORIs screened had mutants with variable expression levels that significantly increased or decreased GFP output relative to WT levels, indicating that the selection of ORI variants can be used to dynamically tailor transient expression levels up to 28-fold for a given ORI. The ability for mutants to increase transient expression in an additional strain of *A. tumefaciens* was also validated using the strain GV3101 and RK2. Within *N. benthamiana*, mutants that were assayed within GV3101 were found to increase or decrease transient expression levels in the same manner as the EHA105 strain (Supplementary Fig. 6). The fold-change difference for EHA105, however, was found to be larger than for GV3101, perhaps because of the hypervirulent nature of EHA105. Superior mutants were also tested in stable transformation systems using GV3101 for *A. thaliana* and EHA105 for *R. toruloides*, with both improving stable transformation, as discussed later in the manuscript. This demonstrates the utility of these mutants in two commonly used strains of *A. tumefaciens*. Additionally, top-performing mutants, along with their WT form, were assayed in EHA105 in another transient expression system, *Lactuca sativa* (Supplementary Fig. 6), and superior mutants increased GFP output for RK2 and pSa. Taken together, these results demonstrate that engineering the binary vector ORI can enable user-controlled manipulation of T-DNA transfer, refining our control over AMT in a transient system.

### Growth rate and copy number impact on AMT is origin specific

**Fig. 4 |:**
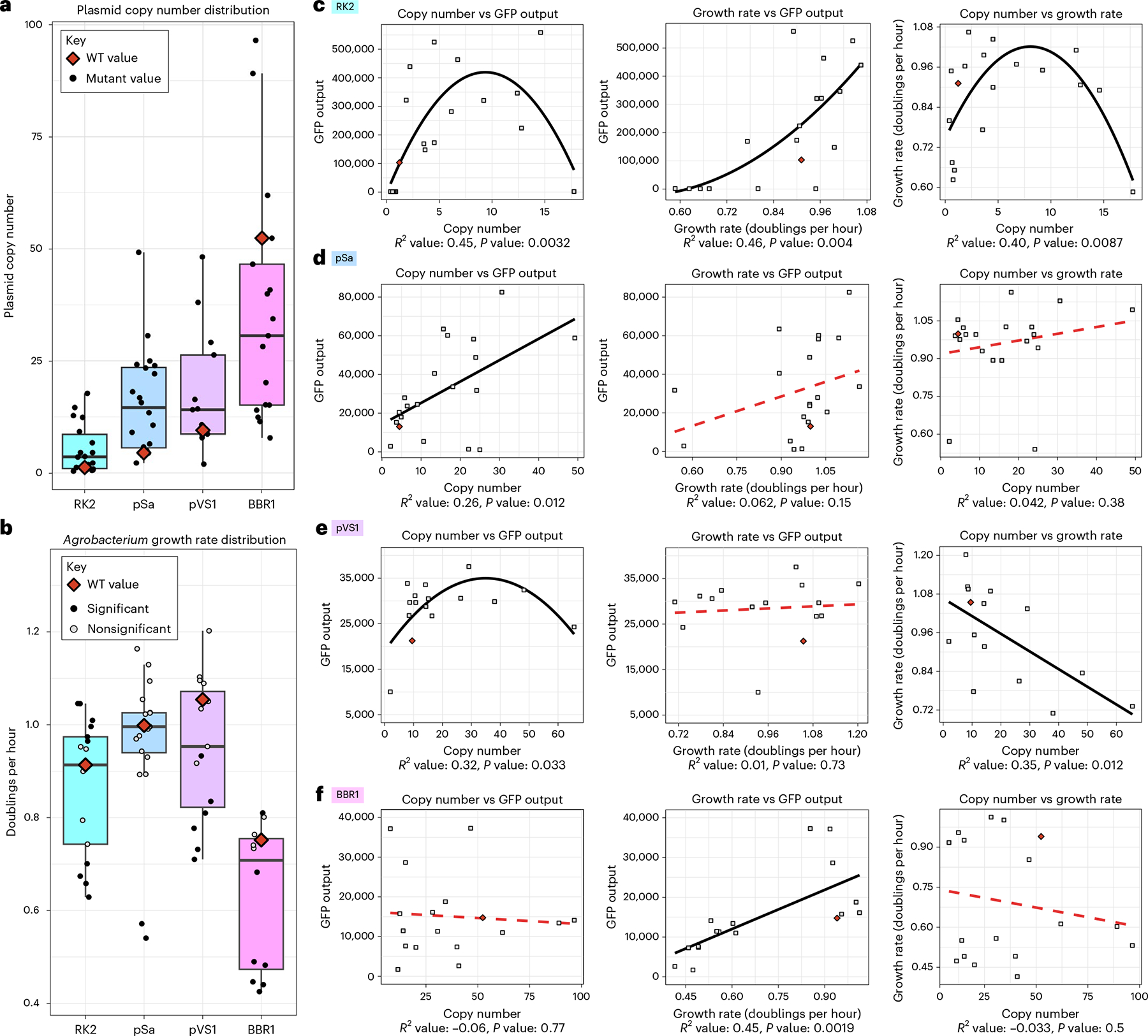
Relationship among copy number, *Agrobacterium* growth rate and transient transformation. **a**, A box plot depicting the measured copy numbers of all mutants and the WT for each ORI as measured by dPCR. Each point represents the average of three biological replicates (full data in Supplementary Fig. 7). The WT copy number is depicted as a red diamond. **b**, A box plot depicting the biological triplicate average of the growth rate for every mutant and WT form of each ORI. The WT growth rates are depicted as red diamonds and mutant growth rates are depicted as circles. These circles are shaded according to the significance of the difference in growth rate from the WT as calculated by a Student’s two-sided *t*-test, using Benjamini–Hochberg correction for multiple testing. Circles are shaded black for *P* < 0.05 and gray for nonsignificant *P* values. For all box plots, the upper and lower bounds represent the 75th and 25th percentiles of the data, respectively, with the median value depicted as the center line; the whiskers extend up to 1.5× the interquartile range. **c**–**f**, Regressions displaying the relationship between binary vector copy number and GFP output from the *N. benthamiana* assay (left), growth rate and GFP output (middle) and binary vector copy number and growth rate (right) for RK2 (**c**), pSa (**d**), pVS1 (**e**) and BBR1 (**f**). The adjusted *R*^2^ value is displayed beneath each regression along with a *P* value for the regression, as derived from the *F* statistic calculated for a two-sided test. Regressions that were not found to have significant predictive power (*P* > 0.05) are plotted as a dashed red line.

To determine the relationship between binary vector copy number and *N. benthamiana* transient expression efficiency, we used digital PCR (dPCR) to quantify the plasmid copy number of each construct (Supplementary Fig. 7 and Supplementary Table 1). While growing the cultures for copy number quantification, we observed that some samples had notably different growth rates compared to the WT ORIs. Thus, we also quantified the growth rates of *A. tumefaciens* harboring WT or mutant ORIs in a time-course plate growth assay in both Luria–Bertani (LB) and a MOPS minimal medium + glucose (Supplementary Figs. 7 and 8). While previous work postulated that increased binary vector copy numbers would correspond to higher AMT efficiencies because of increased T-DNA delivery to target cells^17^, our results demonstrate a more complex relationship among binary vector copy number, strain growth rate and AMT-mediated GFP output (Fig. 4).

Of the ORIs tested in this study, pSa showed the most direct relationship between plasmid copy number and GFP output. The WT copy number of 4.5 increased to 49 copies per cell and no plateau was observed for the regression line between copy number and GFP output, suggesting that the copy number can be increased further to enhance AMT performance. No significant relationship was observed between copy number and growth rate or between growth rate and GFP output for pSa, making copy number the only measured variable of importance.

Unlike the trend observed for pSa, RK2 exhibited an increase in GFP output with higher copy numbers; however, beyond an optimal level, excessively high copy numbers led to a decline in performance. The WT RK2 copy number of 1.2 increased to a maximum of 18 copies per cell in the I305K mutant but this construct showed a notably lower AMT performance. Intermediate copy numbers (that is, 5–15 copies) showed the best results for RK2, making the optimal copy number for this ORI lower than that of pSa, which demonstrates ORI-specific impacts on AMT that fall outside of copy number alone. The growth rates of RK2 mutants with higher copy numbers exceeded the WT growth rate until around seven copies per cell, after which the growth rates declined. There was a direct correlation between growth rate and GFP output, suggesting a tight relationship among these three variables for this ORI. The measured RK2 WT copy number of 1.2 was substantially lower than the value of 7–10 previously reported in the literature^29,30^. We, thus, conducted a verification experiment to determine the WT copy number using three optical densities (ODs; 0.25, 0.5 and stationary phase) and two extraction kits, and we produced a consistent result of a WT copy number of around one per cell. (Supplementary Fig. 9). As the RK2 ORI has undergone progressive truncations over the decades to remove components that are not absolutely essential for plasmid replication, our quantification pertains to the mini-RK2 variant consisting of only the oriV and the *trfA repA* ORF^28^.

In a similar manner to RK2, increased copy numbers of pVS1 increased GFP output until an optimal number that, if exceeded, saw declines in transient GFP expression. The WT copy number of 9.5 increased to a maximum of 66 copies and copy numbers between 30 and 40 produced the highest GFP outputs. There was a direct negative relationship between copy number and growth rate with high-copy-number mutants growing more slowly on average than the WT ORI. Unlike RK2, however, there was no observed relationship between growth rate and AMT performance, and slower-growing mutants outperformed their WT counterparts.

BBR1 was the only ORI that did not follow our hypothesis, and no relationship was found between plasmid copy number and GFP output. The WT form of BBR1 replicates at a rather high 52 copies per cell in *Agrobacterium* and further increasing this value saw no benefit to AMT performance. Rather than copy number, growth rate was the only measured variable of importance, with faster-growing mutants tending to outperform their slower-growing counterparts. Interestingly, plasmid copy number had no relationship with growth rate, implying that the mutants grew at different rates independent of the copy number of the plasmid. The top-performing mutant, E182V, had a copy number of eight, demonstrating that lower copy numbers can potentially outperform the WT form for BBR1.

We also sought to understand whether mutation fold-change enrichment in our initial screen could predict downstream AMT performance. When we assessed the Pearson’s correlation of mutation enrichment with tobacco GFP, growth rate and copy number, only weak correlations between GFP and enrichment and between copy number and enrichment were found to be significant for the pSa origin alone, suggesting that the fold-change enrichment within the checkerboard selection assay is not predictive of any downstream character (Supplementary Fig. 10).

Taken as a whole, our results demonstrate that increased copy numbers improve AMT efficiencies for three of the four ORIs screened. Excessively high copy numbers likely impose some form of a metabolic burden on a cell as previously described^22^, resulting in deleterious effects for pVS1 and RK2 that would likely emerge for higher copies of pSa. Growth rate of the host bacteria is also an important variable for some but not all ORIs and is the only measured variable of importance for BBR1. The intrinsic differences between ORIs and their interaction with host machinery in vivo likely contribute to the unique relationships observed for each ORI. As the four RepA proteins mutagenized in this study are nonhomologous and likely influence the dynamics of plasmid replication differently from one another, we are not surprised by the distinct relationships among copy number, growth rate and GFP output observed. Our findings demonstrate that there are no unifying principles that can explain how different ORI copy numbers correlate with AMT efficiency, highlighting the need for empirical, in vivo testing of each mutant variant to evaluate what is an otherwise unpredictable impact on AMT performance.

### Mutant ORIs improve plant and fungal stable transformation

**Fig. 5 |:**
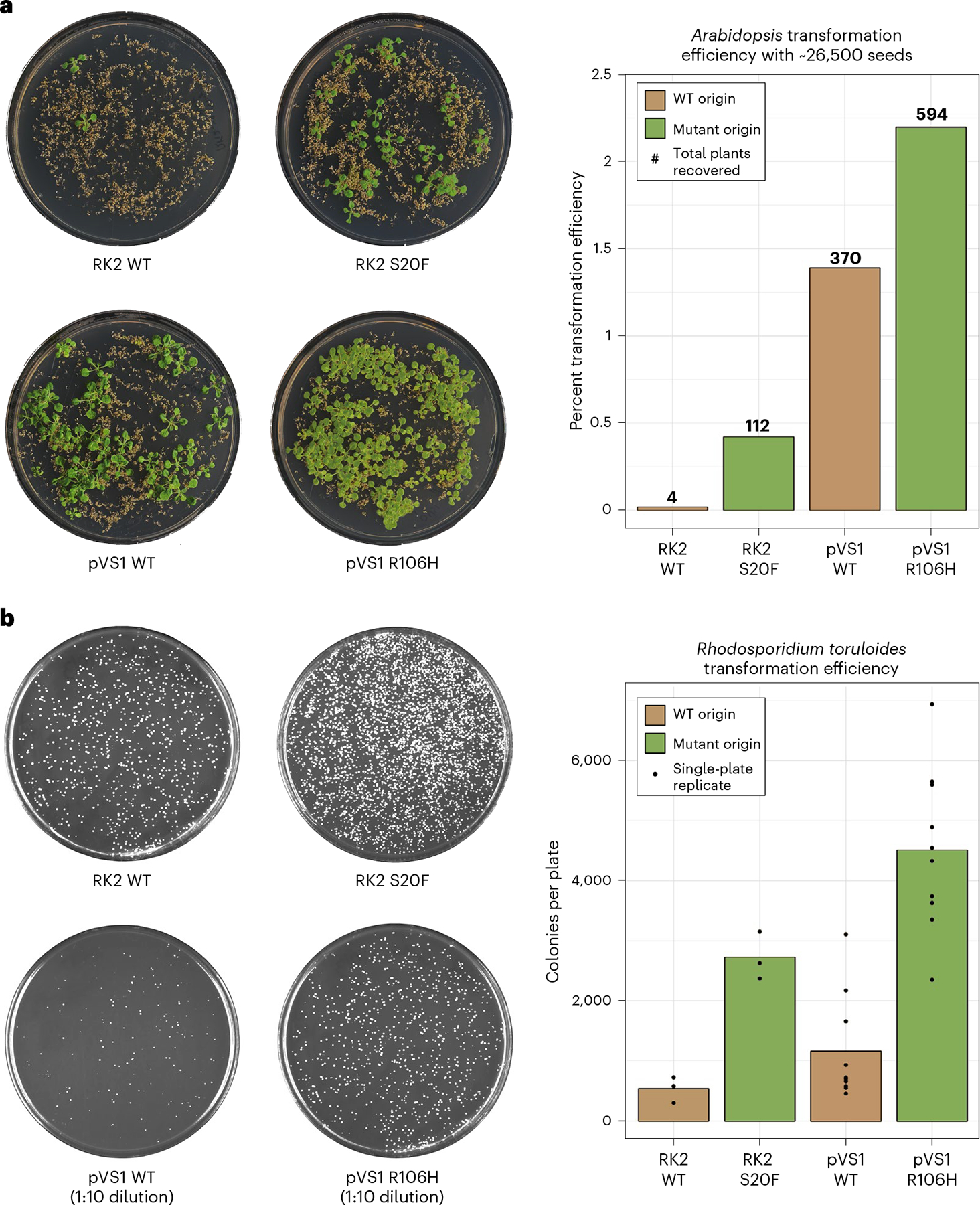
Binary vector origin mutants improve stable transformation of both plants and fungi. **a**, Example plates for the pVS1 and RK2 *A. thaliana* stable transformation experiment. The bar graph reports the observed transformation efficiency (recovered plants/total seeds) with the total number of recovered plants placed above each bar. For both ORIs, the number of recovered plants was significantly higher for the mutant compared to the WT form as computed by a two-sided Fisher’s exact test (RK2, *P* < 2.2 × 10^−16^; pVS1, *P* = 2.97 × 10^−12^). **b**, Example plates for the *R. toruloides* stable transformation experiment. A 1:10 dilution plate is shown for the pVS1 examples because of the high colony density for some R106H mutant plates. The bar graph reports the average number of colonies observed per plate, with individual points representing the colony number per independent transformation replicate. For both ORIs, the number of colonies was significantly higher for the mutant compared to the WT form as computed by a Welch’s two-sided *t*-test (RK2, *P* = 0.0034; pVS1, *P* = 6.49 × 10^−6^).

To evaluate whether the results from the transient tobacco screen could be translated into stable transformation systems for both plants and fungi, stable transformation experiments were conducted for *A. thaliana* and *R. toruloides*. As pVS1 typically achieves the highest transformation efficiency of commonly used ORIs, we selected three mutants and the WT form of pVS1 to determine whether this highly efficient ORI could be improved. Additionally, the top mutant and WT of RK2 and pSa were selected to demonstrate potential improvement in other ORIs. For the *A. thaliana* transformation, 20 plants were floral-dipped per construct and the seeds were bulked into a single stock. A seed weight corresponding to ~26,500 seeds was selected on kanamycin medium and assayed for total plant recovery. The pVS1 R106H mutant increased the *A. thaliana* stable transformation efficiency by 60% over WT pVS1 (Fig. 5). RK2 and pSa had more dramatic increases at 2,800% and 280%, respectively, but with lower total transformation efficiencies than pVS1. The second-highest-expressing and third-highest-expressing pVS1 mutants were also tested and found to increase stable transformation by 35% and 24% (Supplementary Fig. 11).

To further validate this result, a construct delivering a T-DNA conferring hygromycin resistance along with a constitutively expressed *Ruby* reporter was used to floral-dip 20 additional plants for the WT and R106H forms of pVS1. From an additional ~25,000 seeds, the R106H mutant improved the transformation efficiency in this experiment by 103% over WT and the red pigmentation of the plantlets confirmed their status as transgenic (Supplementary Fig. 12).

For the oleaginous yeast *R. toruloides*, the top constructs from pVS1 and RK2 were used alongside their WT counterparts. Both mutants improved the stable transformation efficiency by 390% (pVS1) and 510% (RK2) compared to their WT ORIs. Taken together, these results demonstrate that mutants with increased T-DNA delivery to target cells, as identified in the *N. benthamiana* screen, also have increased stable transformation efficiencies, making *N. benthamiana* a viable platform for rapidly screening ORI mutant impact on AMT.

## Discussion

Using a directed evolution selection assay, this study created and characterized a large library of binary vector copy number variants, surpassing variant levels found for these ORIs in any other bacterial species. As the four ORIs engineered are from unique incompatibility groups, these variants will likely be useful for various prokaryotic engineering efforts. RepA proteins from these ORIs are distinct in their form, regulation and function within the host; therefore, the growth-coupled mutational pipeline is likely amenable to any ORI that replicates with a RepA protein, creating a high-throughput tool to engineer numerous broad-host-range and narrow-host-range plasmids. As plasmid copy number can dramatically impact genetic engineering in bacteria, the libraries of characterized copy number mutants will likely serve to enable more precise genetic control in nonmodel bacteria where no such variants existed previously.

The copy number libraries created in this study enable us to study the impact of binary vector copy number on AMT at a resolution not previously possible. We demonstrated that single SNPs in a binary vector backbone can vary transient expression levels by 28-fold for a given ORI within tobacco, demonstrating a large gradient of T-DNA delivery to target cells. We demonstrated that the results from this tobacco transient expression screen can be translated to improvements in stable transformation efficiency in both plants and fungi. Thus, engineering single SNPs into the binary vector backbone can notably improve AMT efficiency, presenting an easily deployable method for enhancing downstream transformation.

For such transformations, single T-DNA insertions are often desired and previous studies determined that different ORIs that replicate at varying copy numbers alter the average number of T-DNA insertions per cell^18^. This library should enable a user to titrate variable amounts of T-DNA into a cell to find the ideal copy number for both optimized transformation efficiency and desired insertion number. Future work should focus on characterizing the large range of copy numbers (nearly two orders of magnitude) to determine the relationship among binary vector copy number, stable transformation efficiency and quality event generation.

From a gene-editing perspective, the number of repair templates delivered to a cell is thought to limit the efficiency of homology-directed repair (HDR)^35,36^. Transient delivery of increased levels of repair template using a higher-output mutant along with Cas9 or another targeted nuclease may enhance HDR efficiencies, and higher-copy-number mutants from each ORI can be used as tools to evaluate this. Further extensions of this work through stacking individual mutations and exploring different amino acid substitutions at enriched residues should enable an even greater range of copy numbers and AMT performances, providing a route for further optimization. This is particularly true for pSa, which did not have an observed plateau for GFP output with higher copy numbers, suggesting that this number can be raised even further to improve AMT. Taken as a whole, we have created a toolkit of copy number variants derived from single *repA* SNPs that can successfully tailor plant transient expression levels and improve stable transformation in plant and fungal systems, expanding and refining our control over the critical process of AMT.

## Methods

### Media, chemicals and culture conditions

Routine bacterial cultures were grown in LB Miller medium (BD Biosciences). *E. coli* was grown at 37 °C and *A. tumefaciens* was grown at 28 °C shaking at 200 rpm unless otherwise noted. Cultures were supplemented with kanamycin (50 mg L^−1^; Sigma-Aldrich), gentamicin (30 mg L^−1^; Thermo Fisher Scientific), spectinomycin (100 mg L^−1^; Sigma-Aldrich) or rifampicin (100 mg L^−1^; Teknova) when indicated. All other compounds unless otherwise specified were purchased through Sigma-Aldrich.

### Strains and plasmids

All bacterial strains and plasmids used in this work are listed in Supplementary Table 2. All strains and plasmids created in this work are viewable through the public instance of the Joint BioEnergy Institute (JBEI) registry (https://public-registry.jbei.org/folders/847). All strains and plasmids created in this work can be requested from the strain archivist at JBEI with a signed material transfer agreement. All plasmids generated in this paper were designed using Device Editor, which is part of the DIVA suite version 6.1.2 and Open Vector Editor software version 18.3.6, while all primers used for the construction of plasmids were designed using j5 software version 3.4.0 (refs. 37–39). Plasmids were assembled by Gibson assembly using standard protocols^40^, Golden Gate assembly using standard protocols^41^ or restriction digest followed by ligation with T4 ligase^42^. Plasmids were routinely isolated using the QIAprep spin miniprep kit (Qiagen) and all primers were purchased from Integrated DNA Technologies (IDT). Plasmid sequences were verified using whole-plasmid sequencing (Primordium Labs). *Agrobacterium* was routinely transformed by electroporation as described previously using a 1-mm cuvette and a 2.4-kV, 25-μF, 200-Ω pulse^43^.

### Mutating RepA proteins using epPCR

For each origin used in this study, the RepA or RepA-like protein was randomly mutagenized using epPCR^44^. Briefly, the RepA ORFs were amplified with a high-fidelity Phusion polymerase (New England Biolabs (NEB), M0530S) using primers to add BsaI restriction sites to the 5′ and 3′ ends of the product. Bands of the proper size were gel-purified (Zymo Research, D4007) and the resulting product was used as a template. An epPCR master mix was made, containing MnCl_2_ and unequal nucleotide ratios (10 mM TrisCl pH 8.3, 50 mM KCl, 7 mM MgCl_2_, 1 mM deoxycytidine triphosphate, 1 mM deoxythymidine triphosphate, 0.2 mM deoxyadenosine triphosphate, 0.2 mM deoxyguanosine triphosphate, 0.5 mM MnCl_2_, 1 μM forward and reverse primers, 20 pg μl^−1^ purified RepA template and 1 μl of Taq polymerase (Thermo Fisher Scientific, EP0401) per 50-μl reaction). For each reaction, a 12-cycle amplification was run to constrain the mutation number in each product strand to around 1–3 mutations. The appropriate bands were excised and gel-extracted and the resulting product was digested with BsaI and cleaned by column purification (NEB, T1030S)

### Constructing mutant libraries

To make selection vectors, a gentamicin resistance gene (gentamicin-3-acetyltransferase) driven by a salicylic-acid-inducible promoter was originally cloned into pGingerBS-NahR^45^ and then variants were created for each of the pVS1, pSa and RK2 origins using a Gibson-like assembly with NEBuilder HiFi DNA assembly (NEB, E2621L). The entire vector except the RepA protein was then amplified in a Phusion PCR reaction with primers containing PaqCI restriction overhangs designed to have complementary sticky ends with the epPCR products from above. These PCR products were gel-purified and digested with PaqCI before being column-purified and ligated with the digestion product from the epPCRs using T7 DNA ligase (NEB, M0318S).

After 30 min of room temperature incubation, the ligation reaction was column-purified and 1 μl was electroporated into *E. coli* per reaction (NEB, C3020K). Following a 1-h recovery in the supplied medium, the electroporated samples were placed into 50 ml of LB + spectinomycin. A 1:100 dilution was made for each flask and was plated onto solidified LB + spectinomycin to allow for an estimate of library size. Around five flasks were prepared for each origin to create a library size of at least 100,000 independent transformants. After an overnight growth at 37 °C shaking at 200 rpm, 5-ml aliquots from each flask were miniprepped (Qiagen, 27104) and all samples from each origin were combined into single tubes, comprising the mutant vector library of ~100,000 mutants.

Next, 1 μl of these libraries per reaction were then electroporated into *A. tumefaciens* C58C1 electrocompetent cells. Using the same method as above with 50-ml recovery flasks, cells were recovered at 30 °C overnight shaking at 200 rpm and enough flasks were prepared to obtain a library size of around 150,000 mutants. Then, 5 ml of cells from each flask were combined and made into glycerol stocks for use in the higher-copy-number selection screen.

### Selecting higher-copy-number mutants from constructed libraries

A checkerboard assay was used to select for higher-copy-number variants. The mutant libraries described above for each ORI contain *A. tumefaciens* C58C1 cells that each harbor a selection vector comprised of a constitutively expressed spectinomycin resistance gene, a salicylic-acid-inducible gentamicin resistance gene and the ORI of interest with a *repA* gene that was subjected to epPCR to induce random mutations. A 1-ml glycerol stock of each of the mutant libraries was grown in 50 ml of LB + spectinomycin (50 mg L^−1^) overnight at 30 °C shaking at 200 rpm along with a culture of the WT strain for each ORI. Spectinomycin was added to maintain the plasmid within the population during this overnight growth before the actual selection began with gentamicin.

For each ORI, 100 ml of LB + spectinomycin was inoculated with saturated culture from the mutant library or WT control in a 1:200 ratio and 500 μl of this solution was added to each well of a 96-well block. Gentamicin was then added to all wells in the block following a serial dilution row-wise from rows A to H with a dilution factor of 2, starting from 3,000 mg L^−1^ and ending with 23.4 mg L^−1^. Salicylic acid was then added to all wells in the plate following a serial dilution column-wise from columns 1 to 12 with a dilution factor of 2, starting from 5 μM in column 12 and ending with 2.44 nM in column 1. This created 96 unique selection conditions within the block corresponding to different gentamicin and salicylic acid concentrations, enabling selection of the population over a large dynamic range of selection conditions.

The resulting 96-well blocks were then covered in vent film and incubated overnight at 30 °C shaking at 200 rpm. Growth was determined by calculating the OD_600_ of each well the following day using a Genesys 50 (Thermo Fisher Scientific) and the mutant population was compared to its WT counterpart to determine gentamicin–salicylic acid combinations that were lethal to the WT ORI but that permitted growth of the mutant population. Two selection conditions that were WT lethal per ORI were chosen and grown in triplicate in 10-ml cultures of LB + spectinomycin + gentamicin + salicylic acid inoculated with a fresh glycerol stock of the mutant population. A culture containing just WT plasmid was also prepared for each selection condition as a negative control. Additionally, a triplicate of cultures containing the mutant library without gentamicin + salicylic acid selection were grown as an unselected control.

The following selection conditions were used: pVS1, 750 mg L^−1^ gentamicin + 5 μM salicylic acid and 375 mg L^−1^ gentamicin + 156 nM salicylic acid; pSa, 1,500 mg L^−1^ gentamicin + 5 μM salicylic acid and 750 mg L^−1^ gentamicin + 625 nM salicylic acid; BBR1, 2,250 mg L^−1^ gentamicin + 5 μM salicylic acid and 1,000 mg L^−1^ gentamicin + 78 nM salicylic acid; RK2, 1,500 mg L^−1^ gentamicin + 5 μM salicylic acid. The overnight cultures were grown at 30 °C shaking at 200 rpm and plasmid-prepped according to a modified Qiagen protocol (http://www.qiagen.com/us/resources/resourcedetail?id=95083ccb-9583-489e-b215-99bd91c0604e&lang=en).

These samples were then used for the following Tagmentation procedure to barcode the RepA ORFs to allow for identification of enriched mutations within the selected population relative to the unselected control.

### Sequencing mutant library survivors to identify enriched RepA mutations

The extracted plasmids from the selected populations were sequenced using an Illumina MiSeq to identify enriched mutations that may have contributed to their survival in WT-lethal conditions. The entire RepA ORF + 150 bp upstream and downstream was PCR-amplified and gel-purified. Purified samples were then fragmented into random 600-bp pieces and barcoded using the Illumina bead-linked transposon Tagmentation kit (Illumina, 20060059) according to the protocol provided by the manufacturer. The concentration of the barcoded fragments was then measured using a Qubit fluorometer with the Qubit dsDNA HS assay kit (Invitrogen). Library size analysis was conducted using a Bioanalyzer (Agilent Technologies). To enhance the quality of Illumina reads for regions with low variations in the conserved PCR amplicons, 20% PhiX Control v3 DNA (Illumina, FC-110–3001) was added to the library. The libraries were evenly pooled and the library, along with the PhiX mixture at a concentration of 18 pM, was loaded onto the MiSeq platform using the MiSeq Reagent Kit v3 (paired-end, 2 × 300-bp reads; Illumina).

Mapping of Illumina reads was performed as described previously^46^. Briefly, quality control for the reads from this run was performed using FastQC version 0.11.8 and MultiQC version 1.10.1. Read trimming was performed using trimmomatic version 0.39 (ref. 47). Trimmed reads were aligned to their corresponding reference file using Bowtie version 2.4.5 (ref. 48). Mutations in sequencing reads were identified using pysamstats version 1.1.2 and pysam version 0.18.0. All code from this work is publicly available on GitHub (https://github.com/shih-lab/origin_story) and all sequencing reads are available on the National Center for Biotechnology Information (NCBI) Sequence Read Archive (SRA) under BioProject accession PRJNA1031697.

### Building plant GFP expression vectors with enriched RepA mutations from the MiSeq data

To construct GFP expression vectors, promoters of variable strengths derived from the PCONS suite of constitutive plant promoters were used^34^. Expression vectors containing a GFP construct driven by the constitutive plant promoters pCM2 (for RK2 and pSa) or pCL2 (pVS1 and BBR1) along with a 35S::KanR component for stable-line selection were made using Gibson assembly. RK2 was run as a pilot for this project using the stronger pCM2 promoter to drive GFP. Because of the high GFP output of the WT forms of pVS1 and BBR1, a very large fold-change increase in GFP expression could potentially saturate the GFP channel of the plate reader; as such, the weaker pCL2 was used for these origins. As pSa had a weaker starting WT expression, pCM2 was used for this origin. To demonstrate the performance of the highest-performing mutants expressed from the same promoter for all origins, an additional set of vectors were cloned with pCM2::GFP for pVS1 and BBR1 (Supplementary Fig. 6). For the Ruby vector described in Supplementary Fig. 12, the strongly expressing pCH5 promoter was used to drive the *Ruby* reporter^49^.

Single SNPs were introduced into the RepA coding sequence using PCR with the SNP of interest designed into a primer overhang. Proper PCR band lengths were verified in a high-throughput manner using an Agilent ZAG (zero-gel electrophoresis). PCR products were column-purified and assembled using NEBuilder HiFi DNA assembly (NEB, E2621L). The resulting assemblies were transformed by heat shock into XL1-Blue *E. coli* and grown overnight at 37 °C on solidified LB + kanamycin plates. Single colonies were selected, grown in a 4 ml of LB + kanamycin liquid culture and miniprepped; then, the integrity of the plasmid sequence including the proper SNP was verified using whole-plasmid sequencing from Primordium Labs (https://www.primordiumlabs.com/). Verified plasmids were then transformed into the EHA105 strain of *A. tumefaciens* by electroporation and selected on plates of solidified LB + kanamycin (50 mg L^−1^) and rifampicin (100 mg L^−1^) at 28 °C shaking at 200 rpm.

### Screening origin mutants in *N. benthamiana*

A previously established method for transiently expressing genes in *N. benthamiana* was used to screen the impact of the mutant ORIs in planta^50^. Single colonies of transformed EHA105 were selected and used to inoculate 5 ml of LB + kanamycin and rifampicin for overnight growth at 28 °C shaking at 200 rpm. The following morning, 10 ml of fresh LB + kanamycin + rifampicin was added to each vial and the cultures were grown for 2 h. An aliquot from each culture was taken and used to measure the OD_600_. Then, 10 ml of each culture was centrifuged at 3,200*g* for 15 min. The supernatant was removed and cells were diluted to an OD_600_ of 1.0 using an appropriate volume of tobacco infiltration buffer (10 mM MgCl_2_, 10 mM MES and 200 μM acetosyringone (added fresh), pH 5.7). Resuspended cultures were allowed to induce for 2 h at room temperature while gently rocking.

*N. benthamiana* was grown at 25 °C under long-day conditions (16 h of light, 8 h of darkness) of 150 μmol m^−2^ s^−1^ photosynthetically active radiation (PAR; wavelength: 400–700 nm). Sunshine no. 4 growing mixture supplemented with Osmocote was used as the planting medium. For each origin mutant, six 4-week old *N. benthamiana* plants were syringe-infiltrated on the fourth and fifth leaves from the top of the plant. Infiltrated plants were watered and returned to the growth room for 3 days. After this 3-day period, four leaf discs of infiltrated tissue per leaf were taken using a standard 6-mm hole puncher and these discs were placed abaxial side up into a 96-well culture plate filled with 320 μl of water such that the discs were floating on the surface. These 96-well plates were then assayed for GFP fluorescence using a BioTek Synergy H1 microplate reader set for conditions of 483-nm excitation and 512-nm emission. GFP fluorescence readings for each disc were analyzed in R to compare mutant performances compared to the WT origins.

### *L. sativa* transient expression assays

Buttercrunch lettuce plants were grown in 18-cell flats at 25 °C under long-day conditions of 150 μmol m^−2^ s^−1^ PAR using the same Sunshine no. 4 + Osmocote planting medium. Then, 5-week-old plants were infiltrated with a blend of two EHA105 strains consisting of the same GFP binary vector strains as the tobacco experiments and a strain harboring a binary vector for the *P19* gene. *P19* is a viral gene that suppresses plant gene silencing and coinfiltration of this construct aids in transient expression, particularly in more recalcitrant backgrounds^51^. These two strains were grown overnight at 28 °C shaking at 200 rpm and were brought to an OD_600_ of 1.0 and mixed in a 1:1 ratio before inducing in the tobacco infiltration medium mentioned above for 2 h. The fifth leaf of each plant was infiltrated and a 4-day incubation period at 25 °C was used before isolating leaf discs for GFP quantification.

### Copy number quantification

Plasmid copy number was determined using nanoplate dPCR. A QIAcuity EvaGreen PCR kit (Qiagen, 250111) was used for all dPCR reactions. Samples were transferred into an 8,500-partition 96-well QIAcuity nanoplate and loaded into a QIAcuity One system. dPCR reactions were conducted with a standard protocol (2 min at 95 °C followed by 40 cycles of 15 s at 95 °C, 15 s at 56 °C and 15 s at 72 °C). The nanoplate was imaged with an exposure duration of 150 ms and gain of 2. Images were analyzed with the QIAcuity Software Suite. The concentration of plasmids were calculated using Poisson statistical methods by the QIAcuity Software Suite. For each biological replicate, two dPCR reactions were run, one targeting the single-copy *rpoB* gene found within *A. tumefaciens* C58 and one targeting the kanamycin resistance (*KanR*) gene located on the binary vector. The final copy number was determined as the ratio of *KanR* to *rpoB* copies, which served as a measurement of the number of plasmid counts to genomic counts respectively. The following primers were ordered from IDT and used for the dPCR reactions: *KanR* forward, GATCATCCTGATCGACAAGACCGG; *KanR* reverse, CTGCCGAGAAAGTATCCATCATGGC; *rpoB* forward, GAGTACCGGAATCTCGTCAAAGCC; *rpoB* reverse, CGAAGATCTCTACGGCAACTACCTGG.

### Growth rate quantification

The growth rates of all WT and mutant strains were evaluated in both a rich LB medium and a minimal salts medium (MOPS minimal + 10 mM glucose)^52^. For each, a glycerol stock was used to inoculate a 10-ml culture of LB that was grown overnight at 28 °C shaking at 200 rpm. This saturated culture was spun down and resuspended in either MOPS minimal salts + 10 mM glucose or LB medium at a concentration that was 1:200 of the original saturated stock. Next, 150 μl of this bacterial culture was added to each well of a 96-well clear plate in quadruplicate (Falcon, 353072) and the cultures were grown shaking linearly at 1,000 cpm at 28 °C in a BioTek Synergy H1 plate reader. The OD_600_ was measured every 6 min for 24 h; using the data from the plate reader, the growth rate for each culture was calculated using the GrowthCurver package in R. The growth rate in this study is reported as doublings per hour, which is 60 divided by the doubling time.

### *A. thaliana* stable transformation

*A. thaliana* was transformed using the floral dip method of transformation as previously described^53^. *Arabidopsis* seeds were sprinkled over the surface of wet Sunshine no. 4 growing mixture and allowed to grow for 12 days. After this period, 18-pot flats were prepared with wet Sunshine no. 4 growing mixture with Osmocote and five *Arabidopsis* seedlings were transplanted per pot in an X pattern. Flats were grown at 22 °C under short-day conditions (8 h of light, 16 h of darkness) of 150 μmol m^−2^ s^−1^ PAR. After 3 weeks, the flats were moved to a 22 °C chamber with long-day lighting conditions of the same intensity. The first floral meristem from each plant was excised to promote axillary shoot growth. After three more weeks, all plants began flowering with multiple inflorescences and these were used for the floral dip procedure.

The top-performing origin, pVS1, and lower-expressing origins RK2 and pSa were selected for an analysis of impact of enhanced binary vector copy number on stable transformation. The same plasmids used in the tobacco screen were used for this experiment as they contained a 35S::KanR component that allows for the selection of T1 plants with kanamycin. These plasmids were electroporated into the *A. tumefaciens* strain GV3101 and selected on solidified plates of LB + rifampicin, kanamycin and gentamicin grown for 3 days at 30 °C. Then, 300-ml cultures of each strain were grown overnight at 30 °C shaking at 200 rpm in LB + rifampicin, kanamycin and gentamicin. These cultures were centrifuged for 20 min at 3,200*g* and the supernatant was discarded and replaced with 300 ml of floral dip medium consisting of 5% sucrose with 0.02% Silwet (50 g of sucrose and 200 μl of Silwet brought to 1 L with water).

Pots containing five *A. thaliana* plants were gently inverted and the inflorescences were submerged into the *Agrobacterium* solution for 15 s with light agitation. All pots dipped in the same construct were then laid sideways in an empty planting tray and were covered with a plastic lid that had been misted with water. Plants were left at room temperature overnight in this humidity chamber before being returned to an upright position in the 22 °C long-day chamber. Then, 1 week after the initial floral dip, this process was repeated with the same plants to further expose newly grown buds to the bacteria. For each construct, four pots containing a total of 20 plants were dipped; following the second dip, a single stake was placed into one pot and the numerous inflorescences were gently tied together in a single mass. Next, 2 weeks after the second dip, a paper bag was used to cover the inflorescence heaps and the plants were moved to a drying rack to die and desiccate.

Following desiccation, seeds were sifted multiple times to separate them from any silique debris. Then, 200 mg of seeds per sample were measured and placed into tubes for the plating experiment. Seeds were then sterilized by first submerging in 70% ethanol for 1 min followed by shaking in a solution of 50% bleach from a 12.5% sodium hypochlorite stock with a drop of Triton-20 surfactant for 7.5 min before rinsing with sterile water three times. A moderately dense layer of seeds that were distributed in close but not overlapping proximity to each other was spread onto the surface of selection plates consisting of Murashige and Skoog salts + 1 g L^−1^ MES brought to a pH of 5.7 along with 50 mg L^−1^ kanamycin and 200 mg L^−1^ timentin to kill residual *A. tumefaciens* and 8 g L^−1^ agar for solidification. Each 200 mg of seeds approximately covered 6 5-inch plates. The plates were then wrapped in vent tape, placed into a long-day 22 °C chamber and allowed to grow for 3 weeks before quantification of successfully growing nonchlorotic plants.

### *R. toruloides* transformation

To construct a *R. toruloides* expression vector, the plasmid JPUB_013523 (ref. 54) was modified to contain the WT or mutant forms of the pVS1 or RK2 ORIs (R106H and S20F mutants, respectively). Origin variants from this base vector were created using Gibson assembly and were electroporated into the EHA105 strain of *A. tumefaciens*. Single colonies were used to inoculate LB + kanamycin and rifampicin for overnight growth at 28 °C shaking at 200 rpm. AMT of *R. toruloides* was conducted as previously described^55^.

Selection was conducted on plates containing nourseothricin; for each transformation, plates with a 1:10 dilution along with a full-concentration plating of remaining cells were made. Plates were incubated at 30 °C for 2 days and then imaged using a AnalytikJena UVP GelSolo followed by colony count quantification using Fiji (https://imagej.net/software/fiji/downloads).

## Supplementary Material

Figures

Tables

Additional information

**Supplementary information** The online version contains supplementary material available at https://doi.org/10.1038/s41587-024-02462-2.

## Data Availability

Next-generation sequencing data are publicly available from the NCBI SRA under BioProject accession PRJNA1031697. All plasmid sequences used in this study were published on the JBEI public repository (https://public-registry.jbei.org/folders/847). Source data are provided with this paper.
