# Author Correction: Binary vector copy number engineering improves *Agrobacterium*-mediated transformation

**DOI:** 10.1038/s41587-025-02959-4

**Published:** 2025-11-24

**Authors:** Matthew J. Szarzanowicz, Lucas M. Waldburger, Michael Busche, Gina M. Geiselman, Liam D. Kirkpatrick, Alexander J. Kehl, Claudine Tahmin, Rita C. Kuo, Joshua McCauley, Hamreet Pannu, Ruoming Cui, Shuying Liu, Nathan J. Hillson, Jacob O. Brunkard, Jay D. Keasling, John M. Gladden, Mitchell G. Thompson, Patrick M. Shih

**Affiliations:** 1 Joint BioEnergy Institute, Emeryville, CA USA; 2 Environmental Genomics and Systems Biology Division, Lawrence Berkeley National Laboratory, Berkeley, California USA; 3 Department of Plant and Microbial Biology, University of California, Berkeley, Berkeley, CA USA; 4 Department of Bioengineering, University of California, Berkeley, Berkeley, CA USA; 5 Biological Systems and Engineering Division, Lawrence Berkeley National Laboratory, Berkeley, CA USA; 6 Laboratory of Genetics, University of Wisconsin-Madison, Madison, WI USA; 7 Sandia National Laboratories, Livermore, CA USA; 8 Department of Chemistry, University of California, Davis, Davis, CA USA; 9 Department of Chemical and Biomolecular Engineering, University of California, Berkeley, Berkeley, CA USA; 10 QB3, University of California, Berkeley, Berkeley, CA USA; 11 Center for Biosustainability, Danish Technical University, Kongens Lyngby, Denmark; 12 Innovative Genomics Institute, Berkeley, CA USA

**Keywords:** Molecular engineering in plants, Bacterial genetics

Correction to: *Nature Biotechnology* 10.1038/s41587-024-02462-2, published online 4 November 2025.

In the version of the article initially published, in Fig. 5b, the “pVS1 R106H (1:10 dilution)” image was a duplicate of the “RK2 WT” image. Fig. 5b has now been amended with the correct “pVS1 R106H (1:10 dilution)” image in the HTML and PDF versions of the article. Additionally, the Supplementary information has been updated to include Supplementary Fig. 13, which displays raw sample plate images from across the dataset.

