# Supplementary material for: Binary vector copy number engineering improves Agrobacterium-mediated transformation
Source: Nat Biotechnol. 2024 Nov 4;43(10):1708–16. doi: 10.1038/s41587-024-02462-2 (PMC12147919; doi:10.1038/s41587-024-02462-2)
Supplement: Supplementary file 1 — Supplementary Figs. 1–12. [file 41587_2024_2462_MOESM1_ESM.pdf]

nature biotechnology

Supplementary information https://doi.org/10.1038/s41587-024-02462-2

Binary vector copy number engineering improves *Agrobacterium*-mediated transformation

In the format provided by the authors and unedited

Supplementary Information - Table of Contents

	**Page**
**Supplementary Figure 1:** RepA proteins from BBR1, pSa, pVS1, and RK2 are non-homologous and are derived from distinct ORIs.	**2**
**Supplementary Figure 2:** Checkerboard assays find unique selection conditions for each ORI that are WT lethal but permissible for growth within the mutant libraries.	**3**
**Supplementary Figure 3:** RepA mutagenesis across the full length of 4 distinct RepA proteins.	**4**
**Supplementary Figure 4:** Mutant enrichment analysis finds distinct enriched residues within the selected checkerboard assay population for each RepA protein.	**5**
**Supplementary Figure 5:** Full Length AlphaFold models with PLDDT scores.	**6**
**Supplementary Figure 6:** Comparison of standardized GFP expression of best origin mutants.	**7**
**Supplementary Figure 7:** Plasmid copy number and growth rate quantification.	**8**
**Supplementary Figure 8:** Growth rate comparison between LB and minimal media.	**9**
**Supplementary Figure 9:** Copy number validation of RK2 origin.	**10**
**Supplementary Figure 10:** Correlation between copy number, growth rate, transient expression output and mutant enrichment in initial selection.	**11**
**Supplementary Figure 11:** Top mutants for the RK2, pSa, and pVS1 ORIs enhance *Arabidopsis* stable transformation efficiency relative to their respective WT ORI.	**12**
**Supplementary Figure 12:** Validation of *A. thaliana* transformation improvement with the pVS1 R106H mutant using a Ruby construct.	**13**
**Supplementary Table 1:** ORI mutant copy number, growth rate, checkerboard selection enrichment, and GFP transient expression data in *N. benthamiana*	**Separate file**
**Supplementary Table 2:** Strains and constructs utilized	**Separate file**

a)

```
pSa_RepA        MPKNNKAPGHRINEIIKTSLALEMEDAREAGLVGYMARCLVQATMPHTDPKTSYFERTNG
RK2_RepA        ---MNRTFDRKAYRQELIDAGFSAEDAETIASRTVMRAPR-ET---FQSVGSIVQQATAK
BBR1_RepA       --------------------------MEISMATQSREIGIQA---KNKPGH-W------
pVS1_RepA       ------VSGRKPSGPVQIGAALGDDLVEKLKAAQAAQRQRIEA---EARPGESWQAAADR
                                                         ::

pSa_RepA        IVTLSIM-GKPSIGLPYGSMPRT---------LLAWICTE-----------AVRTKDPVL
RK2_RepA        IERDSVQLAPPALPAPSAAVERSRRLEQEAAGLAKSMTIDTRGTMTTKKRKTAGEDLAKQ
BBR1_RepA       VQTERKA--HEAWAGLIARKP-------TAAMLLHHLVAQ------------MGHQNAVV
pVS1_RepA       IRKESRQ--PPAAGAPSIRKPPK---GDEQPDFFVPMLYD------------VGTRDSRS
                :          :                    :   :  :                 .

pSa_RepA        NLGRSQSEFLQRLGMHTDGRYTATLRN-----------QAQRLFSSMISLA---------
RK2_RepA        VSEAKQAALLKH-TKQQIKEMQLSLFD------------IAPWPDTMRAMPNDTARSALF
BBR1_RepA       VSQKTLSKLIGR-SLRT---VQYAVKD----------LVAERW------IS---------
pVS1_RepA       IMDVAVFRLSKR-DRRAGEVIRYELPDGHVEVSAGPAGMASVWDYDLVLMA---------
                        :  .   .        : :                      :.

pSa_RepA        -------------------------GEQGNDFGIENVVIAKRAFLFWNPKRPED-----
RK2_RepA        TTRNKKIPREALQNKVIFHVNKDVKITYTGAELRADDDELVWQQVLEYAKRTP--IGEPI
BBR1_RepA       ---------------------------------VVKL------------NGPGTVSAYV
pVS1_RepA       ---------------------------------VSHLTESMNRYREGKGDKPGRVFRPH
                                                    *

pSa_RepA        ---------RALW---DSTLTLTGDFFEEVTRSPVPIRIDYLHAL------RQSPLAMDI
RK2_RepA        TFTFYELCQDLGWSINGRYYTKAEECLSRLQATAMGFTSDRVGHL-------ESVSLLHR
BBR1_RepA       VND------RVAW---GQPRDQL-----RLSVFSAAVVVDHDDQD---------ESLLGH
pVS1_RepA       VADVLKFCRRADG---GKQKDDLVETCIRLNTTHVAMQRTKKAKNGRLVTVSEGEALISR
                                .            :      .                    :

pSa_RepA        YTWL-----TYRVFLLRAKGRPFVQIPWVALQAQFGSSYGSRARNSPELDDKARERAERA
RK2_RepA        FRVLDRGKKTSRCQVLIDEEIVVLFAGDHYTKFIWE-KYRKLSPTARRMFDYFSSHREPY
BBR1_RepA       GDLR-------RIPTLYPGEQQLPTG--------PG-EEP---PSQPGIPGMEPD-----
pVS1_RepA       YKIV-------KSETGRPEYIEIELADWMYREITEG-KNPDVLTVHPDYFLIDPG-----
                           .          .               .

pSa_RepA        ALASFKYNFKKRL-----------------------------REVLIVYPEASDCIEDD
RK2_RepA        PLKLETFRLMCGSDSTRVKKW----------------------REQVG---EACEELR-G
BBR1_RepA       -L----------PALTETEEW------ERRG------------QQRLPMPDEPCF-LDDG
pVS1_RepA       -IGRFLYRLARRAAGKAEARWLFKTIYERSGSTGEFKKFCFTVRKLIGSNDLPEYDLKEE
                 :                                      .: :        .   :

pSa_RepA        G---ECLRIKSTRLHVTRAPGKGARIGPPPT
RK2_RepA        SGLVEHAWVNDDLVHCKR-------------
BBR1_RepA       EPLEPPTRVTLPRR-----------------
pVS1_RepA       AGQAGPILVMRYRNLIEGEASAGS-------
                        :
```

b) BLASTp results for RepA proteins

Protein 1	Protein 2	E-value
RK2_RepA	pSa_RepA	12
RK2_RepA	pVS1_RepA	6.2
RK2_RepA	BBR1_RepA	7.4
pSa_RepA	pVS1_RepA	>15
pSa_RepA	BBR1_RepA	>15
pVS1_RepA	BBR1_RepA	>15

Proteins with an E-value above 1 are unlikely to be related. The RepA proteins used in this study are all distinct and unrelated.

Supplementary Figure 1: RepA proteins from BBR1, pSa, pVS1, and RK2 are non-homologous and are derived from distinct ORIs. Despite all being named "RepA" (or alternatively TrfA for RK2), these 4 proteins share no homology with one another. RepA is a general name given to trans-acting factors that help mediate plasmid replication, regardless of their evolutionary origin, and as we have selected 4 unrelated ORIs from unique incompatibility groups, these proteins are non-homologous. An attempted alignment of the primary sequence of the 4 RepA proteins is shown in **(a)**. The E-value of each potential pairing of the proteins is shown in **(b)** and was conducted using a BLASTp search. All comparisons were found to be notably above 1 which is a general threshold for denoting a potential relation.

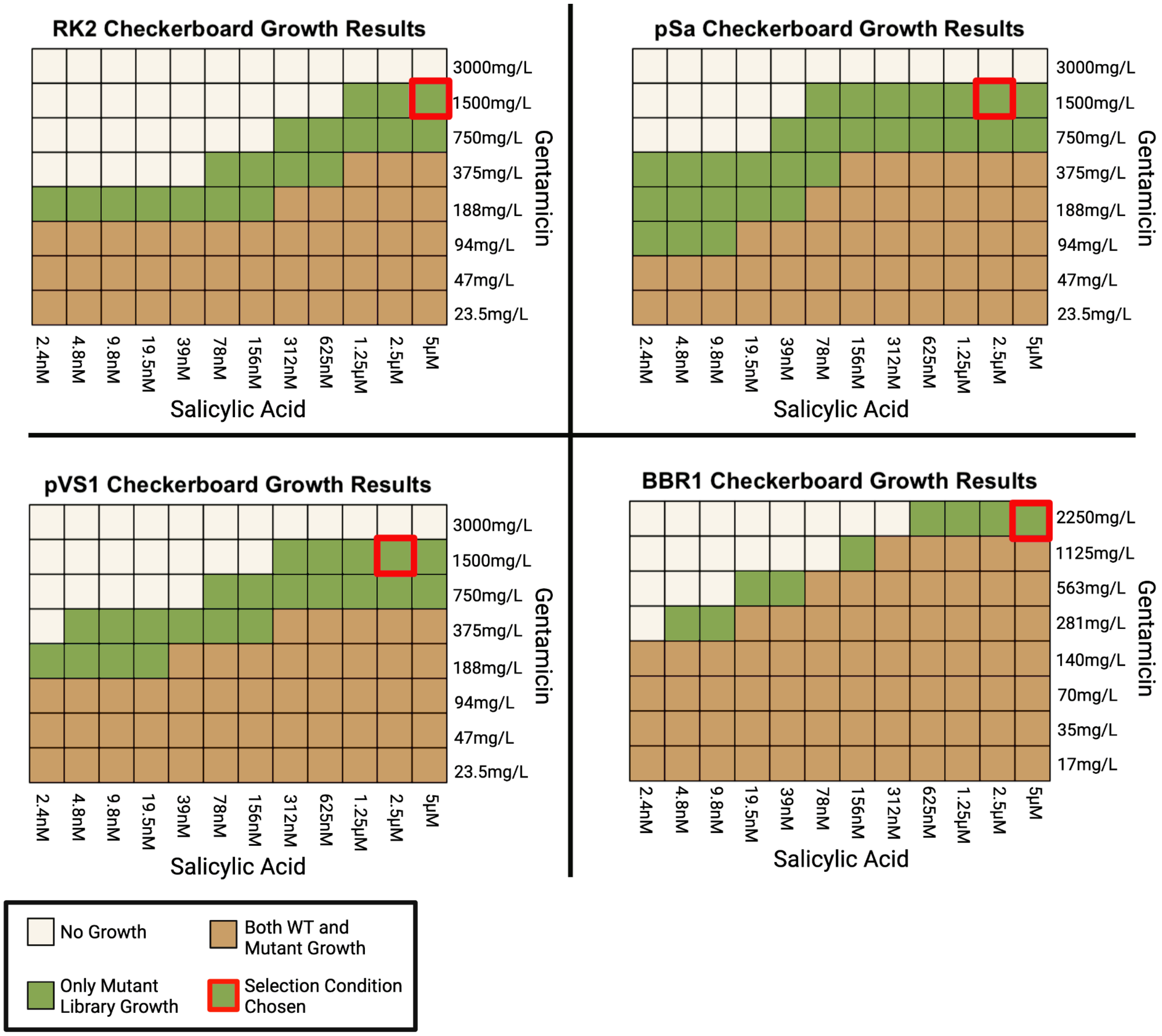

Supplementary Figure 2: Checkerboard assays find unique selection conditions for each ORI that are WT lethal but permissible for growth within the mutant libraries. Depicted are the growth results from the checkerboard assays conducted for the 4 ORIs. For each ORI, 2 96-well blocks were grown for the WT strain and the mutant library. Each plot represents a superimposition of the wells that permitted bacterial growth. Wells that permitted growth for both the WT and mutant library are shown in brown. Wells that only grew for the mutant library and that were lethal for the WT strain are depicted in green. For each ORI, the selection condition that was used for sequencing the population is outlined in red.

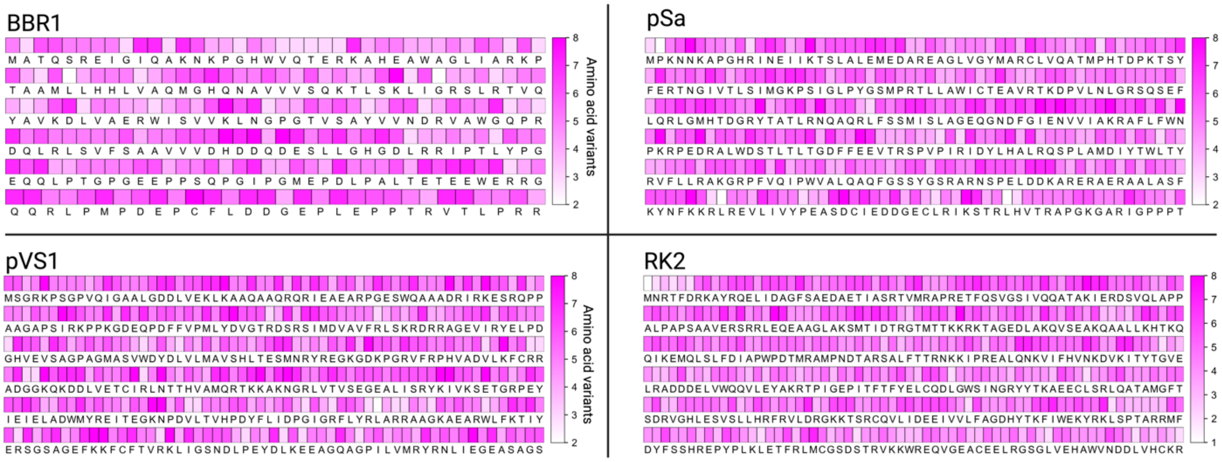

Supplementary Figure 3: RepA mutagenesis across the full length of 4 distinct RepA proteins. Depicted are heatmaps of the 4 RepA proteins showing the number of amino acid variants found per primary sequence position. Each box corresponds to a single residue within the RepA protein for the specified ORI. Letters beneath each box correspond to the WT residue identity in each protein.

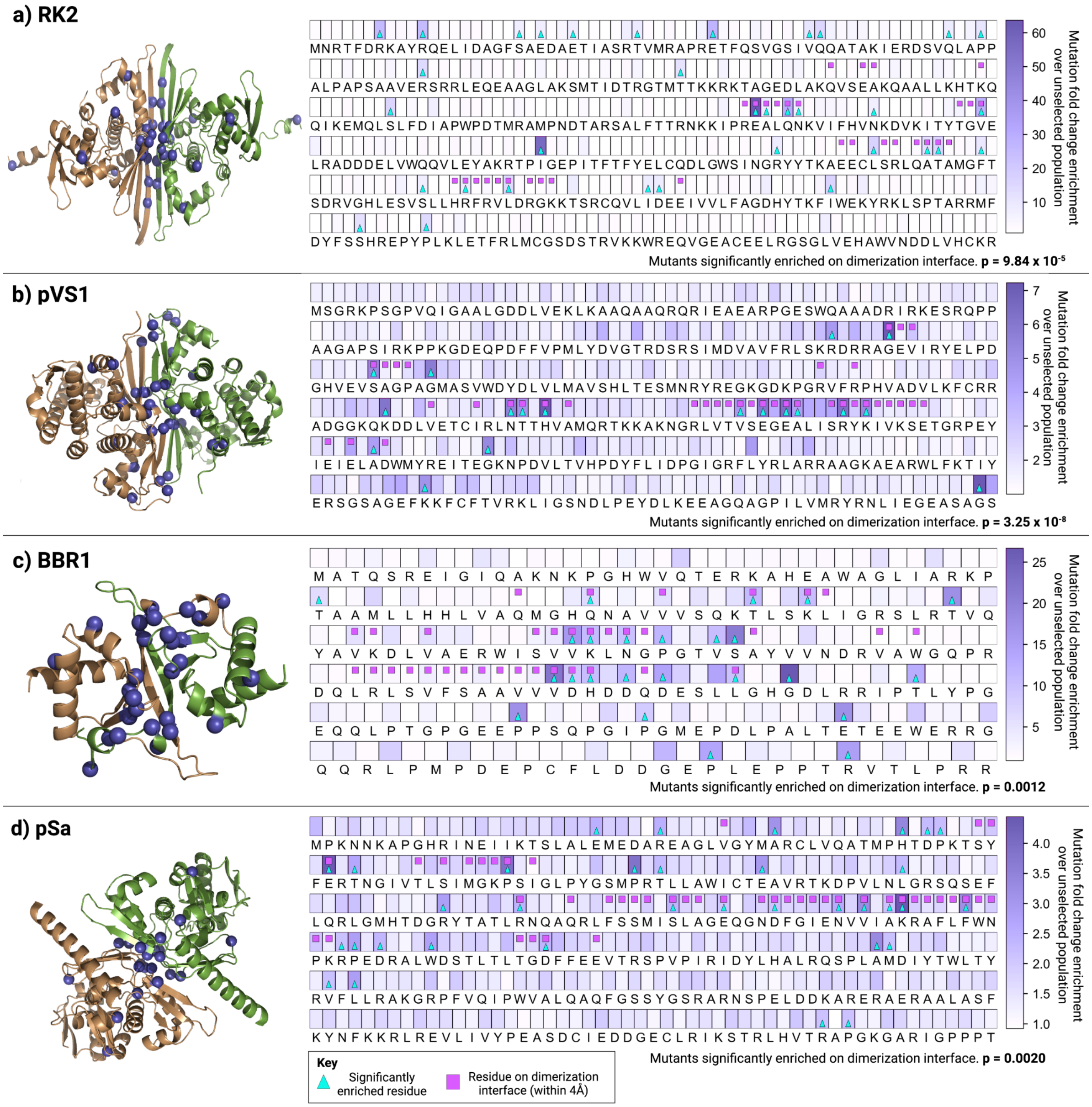

Supplementary Figure 4: Mutant enrichment analysis finds distinct enriched residues within the selected checkerboard assay population for each RepA protein. A heat map for each residue of each RepA protein from the 4 ORIs is shown. Intensity of the shading corresponds to the degree of mutation enrichment at that codon in the selection conditions used from the checkerboard assay vs the unselected mutant population. Codons that were under heavy positive selection are more heavily shaded. Residues that met the cutoff threshold for each ORI have a cyan triangle and comprise the mutants used in this study. Residues that fall on the putative dimerization interface as calculated by any residue within 4 Å of the partner monomer in the AlphaFold model are marked with a green square. A hypergeometric test was conducted for each RepA protein to determine if selected mutation sites were enriched on the dimerization interface, and

the p-values for this test are reported. All RepA proteins were determined to be significantly enriched

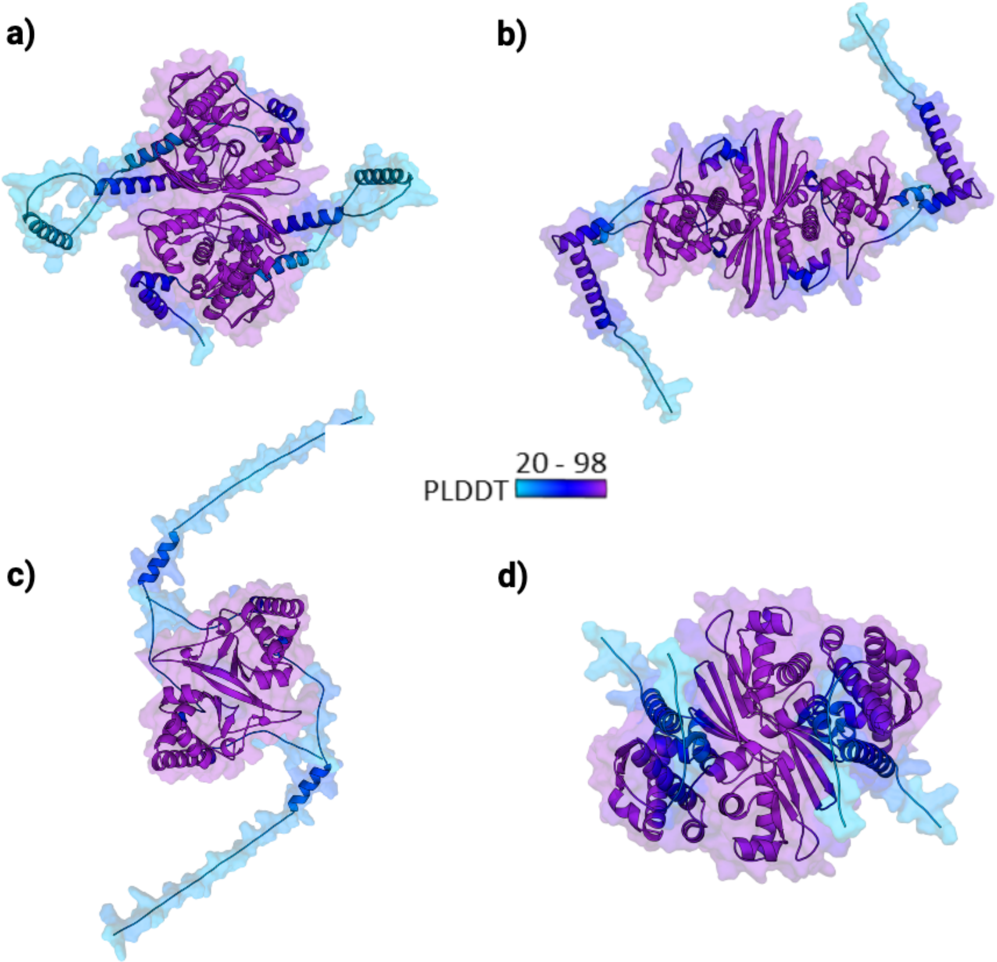

Supplementary Figure 5: Full Length AlphaFold models with PLDDT scores. Depicted are full length AlphaFold modeled dimers of RK2 (**a**), pVS1 (**b**), BBR1 (**c**), and pSa (**d**). Models are shaded according to their PLDDT scores for each residue corresponding to the predicted modeling quality. All 4 RepA proteins have well-modeled cores around the predicted dimerization interface with poorly-modeled/unstructured C and N-terminal tails.

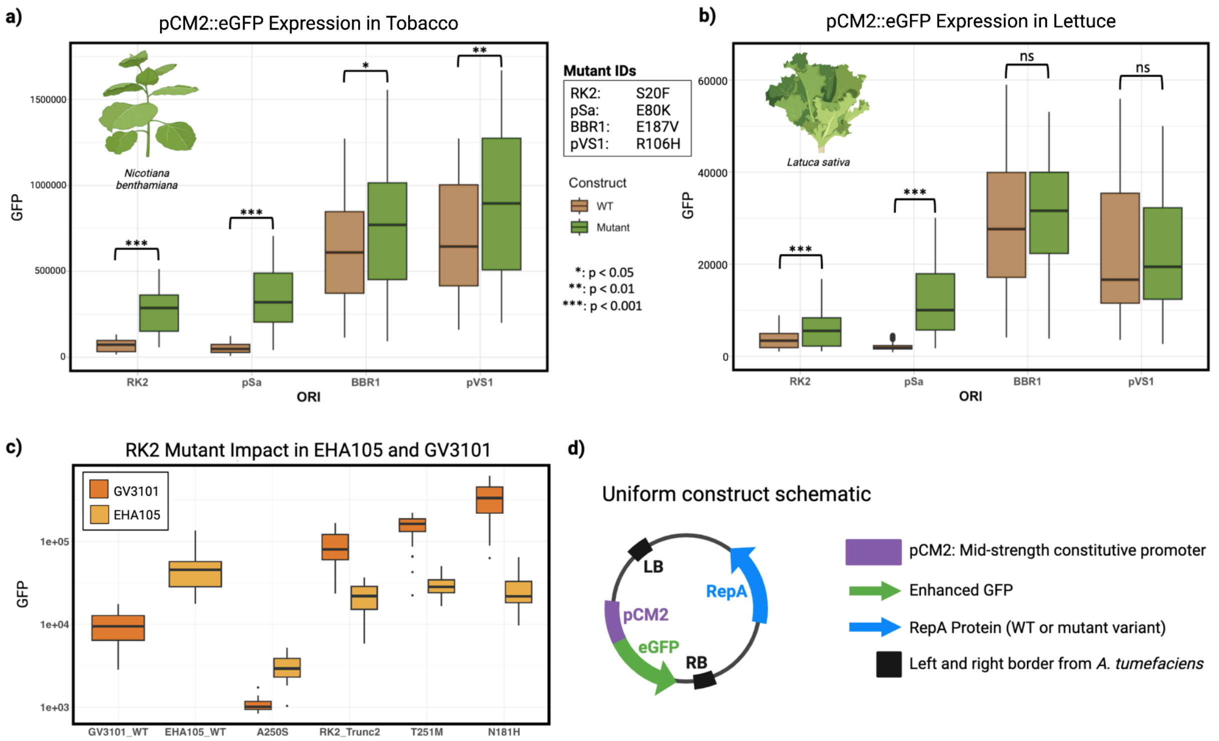

Supplementary Figure 6: Comparison of standardized GFP expression of best origin mutants. A standardized expression vector consisting of the constitutive pCM2 promoter driving eGFP was constructed for each WT ORI and the highest expressing mutant found in the *N. benthamiana* assay. A comparison of the WT and mutants for each ORI is shown in both *N. benthamiana* (**a**) and *Lactuca sativa* (**b**) (N = 48 leaf disks from 6 plants). All mutants were significantly higher expressing in *N. benthamiana* compared to their WT forms. The mutant forms of RK2 and pSa were significantly higher expressing in *Lactuca sativa* while the increases observed for pVS1 and BBR1 were not significantly different in this experiment. A subset of RK2 mutants with variable expression levels along with the WT form were assayed in *N. benthamiana* using the EHA105 and GV3101 strains of *A. tumefaciens* to determine if the mutant ORIs could function in another *A. tumefaciens* strain (**c**). The significant increase/decrease observed in EHA105 relative to WT RK2 was mimicked in the less virulent GV3101 strain. For all boxplots, the upper and lower bounds represent the 75th and 25th percentile of the data, respectively, with the median value depicted as the center line, and the whiskers extend up to 1.5x the interquartile range. Points that are beyond the 1.5x interquartile range are plotted. A schematic of the uniform vector used is displayed in (**d**).

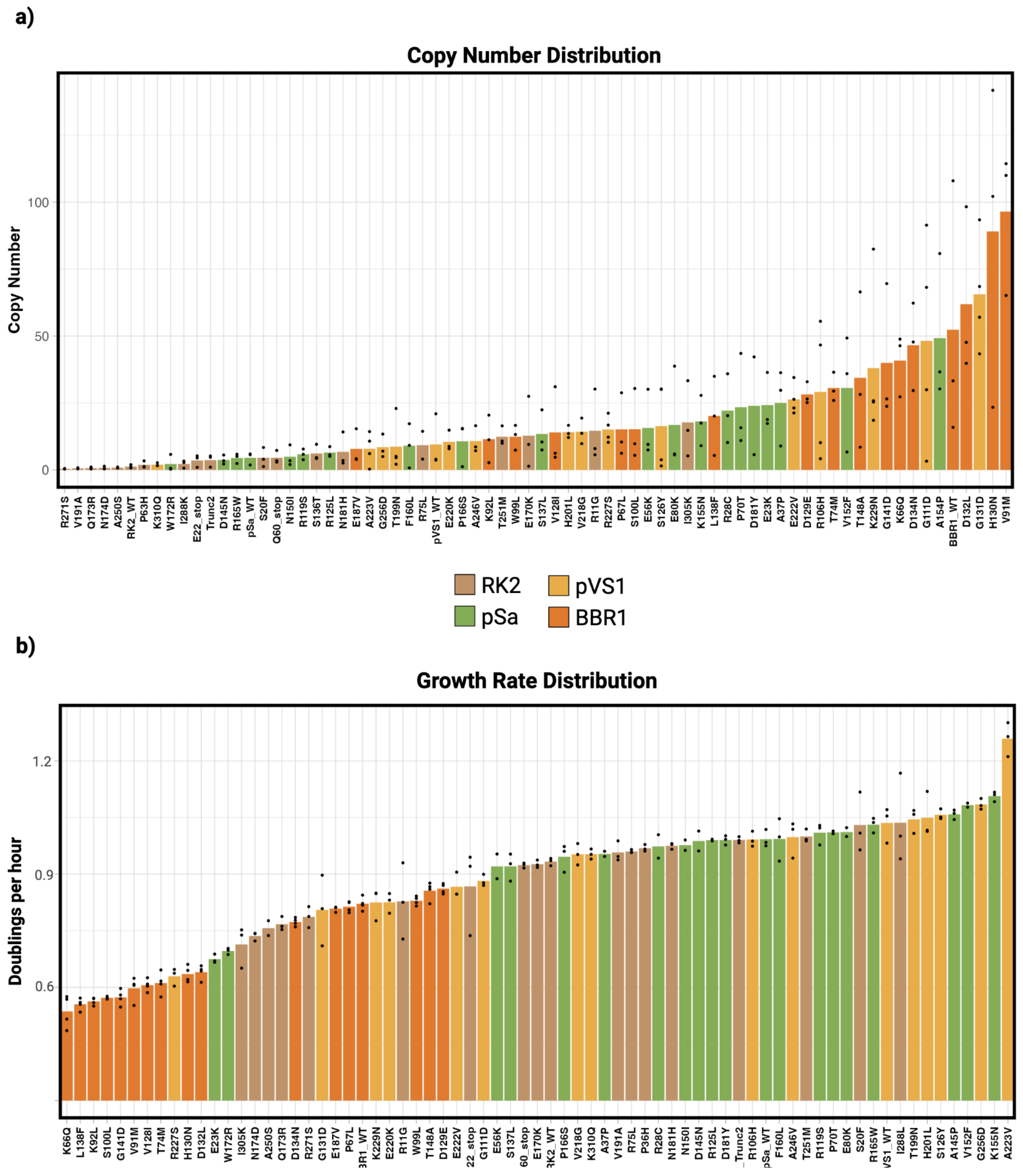

Supplementary Figure 7: Plasmid copy number and growth rate quantification. An ordered bar graph depicting the quantified copy numbers (**a**) and growth rates (**b**) of each mutant and WT origin. For each, the individual copy number or growth rate calculated for each biological replicate (N=3) is depicted as a black dot. Bars are shaded according to their origin.

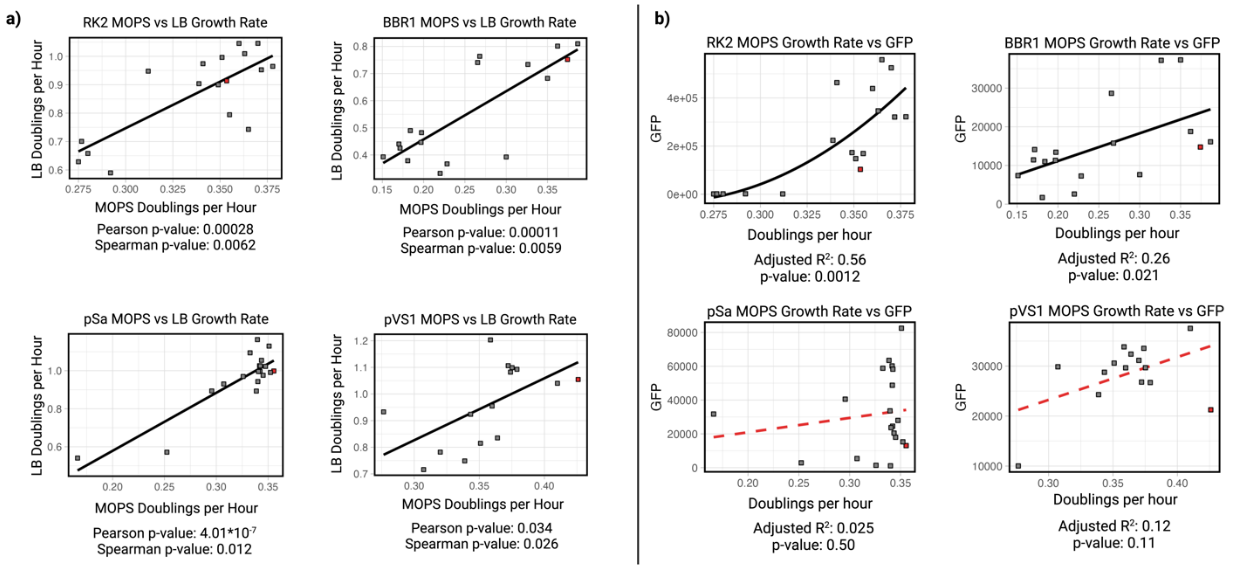

Supplementary Figure 8: Growth rate comparison between LB and minimal media. To evaluate potential differences in growth between a rich LB medium mimicking culture conditions before AMT infection and a minimal salts medium, MOPS minimal salts + 10mM glucose, all mutants and their WT counterparts were grown in quadruplicate in a plate growth assay. Both Pearson and Spearman correlations were computed for these regressions, and their significances were calculated using the t-statistic. These p-values are depicted beneath each regression. For all ORIs, no significant differences in the growth trends were found for the regressions between LB and MOPS minimal growth rate as measured in doublings per hour ($p > 0.05$) (**a**). The relationship between the MOPS minimal growth rates and variant GFP outputs in the *N. benthamiana* assay is shown in **(b)**, and the Adjusted R^2 value is displayed beneath each regression along with a p-value for the regression as derived from the F-statistic for a two-sided test. Regressions that were not found to have significant predictive power ($p > 0.05$) were plotted as a dashed red line.

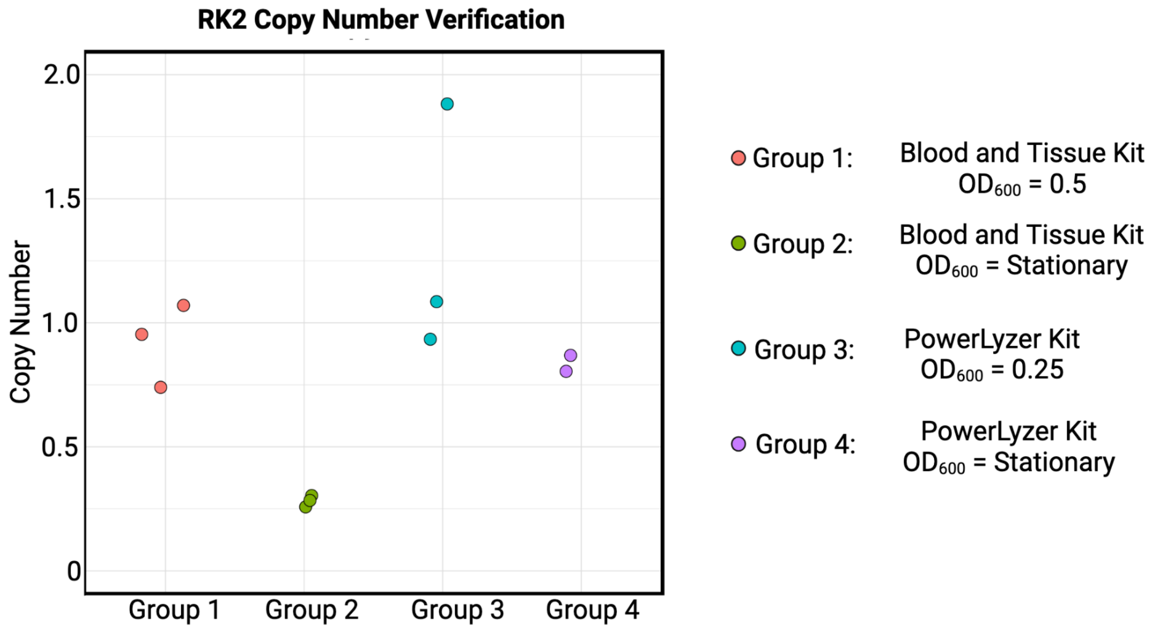

Supplementary Figure 9: Copy number validation of RK2 origin. As our measured copy number for WT RK2 differed from values previously reported in the literature, a verification experiment was conducted using 2 total DNA extraction kits (Qiagen Blood and Tissue and Qiagen Powerlyzer) with both stationary and log phase growing *A. tumefaciens*. A 26,000 well partition plate was used for greater data resolution, and as there are only 24 wells on this plate, a single replicate was omitted from Group 4 to leave room for a negative control for both primer pairs. These results confirm with high confidence that the RK2 ORI used in this study maintains a copy number of around 1 during active growth.

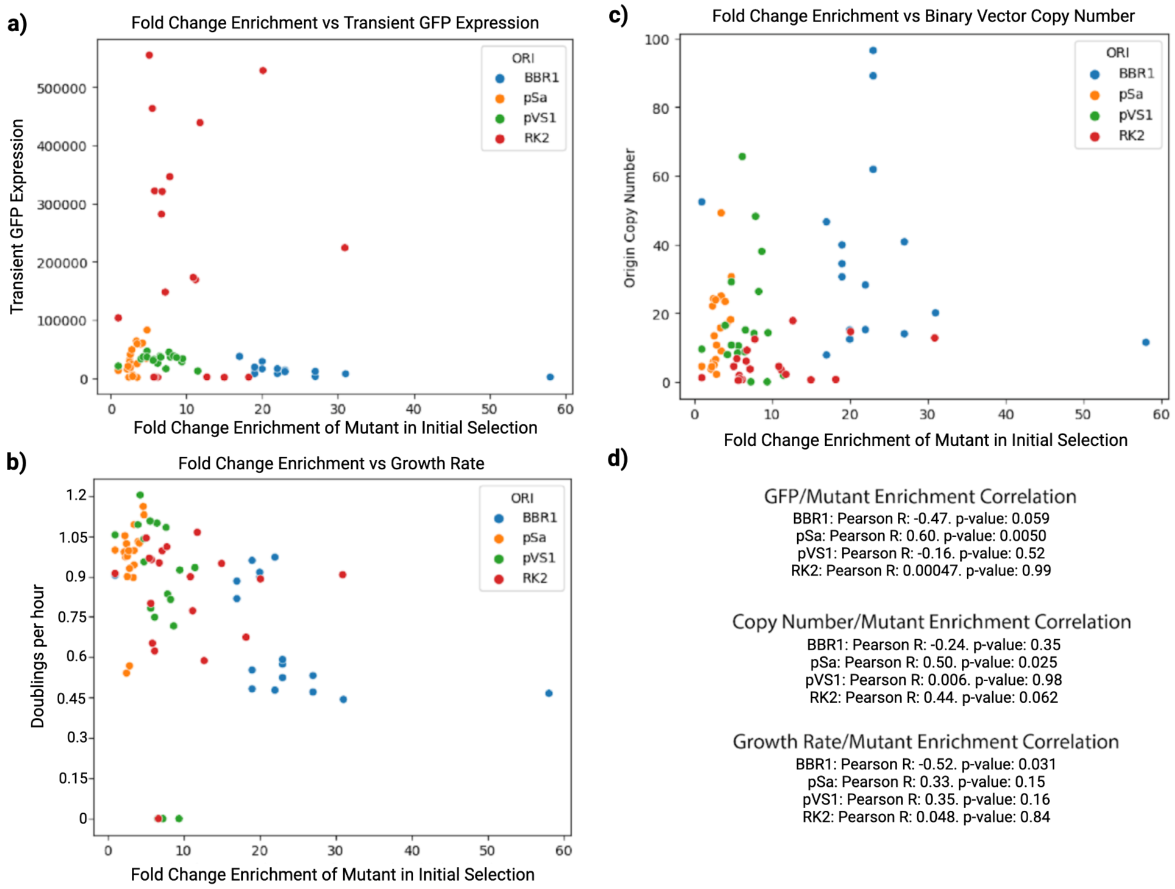

Supplementary Figure 10: Correlation between copy number, growth rate, transient expression output and mutant enrichment in initial selection. The relationship between fold change enrichment of mutant residues from analyzed NGS data and tobacco transient expression output (**a**), strain growth rate (**b**), and copy number (**c**) are plotted. The Pearson R statistic and p-value for a two-sided test of each regression are reported in (**d**).

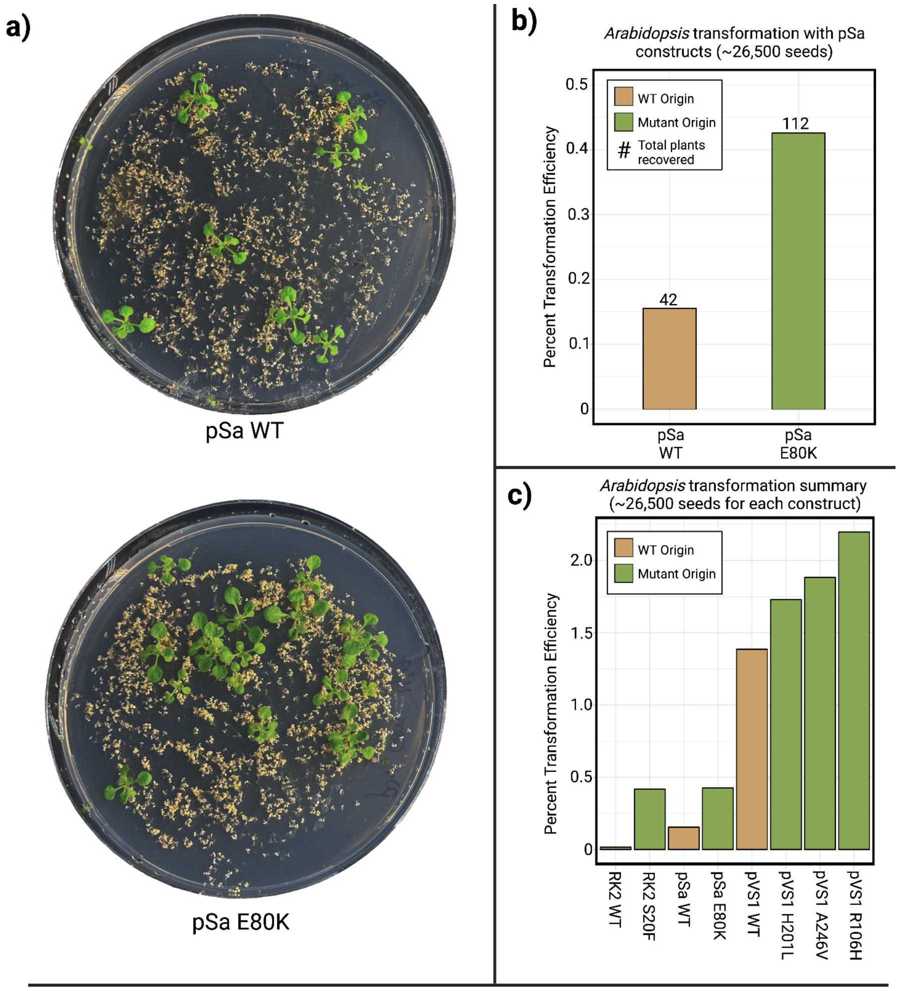

d)

Origin	Construct	Seed planted (mg)	Seed Number	Plants Transformed	Transformation Efficiency (%)
pVS1	WT	534	26700	370	1.39
pVS1	R106H	541	27050	594	2.20
pVS1	A246V	525	26250	494	1.88
pVS1	H201L	537	26850	464	1.73
RK2	WT	526	26300	4	0.02
RK2	S20F	537	26850	112	0.42
pSa	WT	544	27200	42	0.15
pSa	E80K	532	26600	113	0.42

Supplementary Figure 11: Top mutants for the RK2, pSa, and pVS1 ORIs enhance *Arabidopsis* stable transformation efficiency relative to their respective WT ORI.. The WT and E80K mutant of pSa were also assayed in *A. thaliana* using the same vectors as the tobacco transient expression screen, and the results of this experiment are shown. **a,** an example plate from the pSa transformation for both WT and E80K **b,** quantification of recovered plants from ~26,500 planted seeds along with the calculated transformation efficiency **c,** summary of all transformation results for *A. thaliana* including for 2 additional pVS1 mutants along with the top-performing R106H mutant. **d,** a summary table of the *A. thaliana* experiments. As a slightly different seed weight was used for each construct, the estimated seed number for each is shown. This number was used to calculate the transformation efficiency.

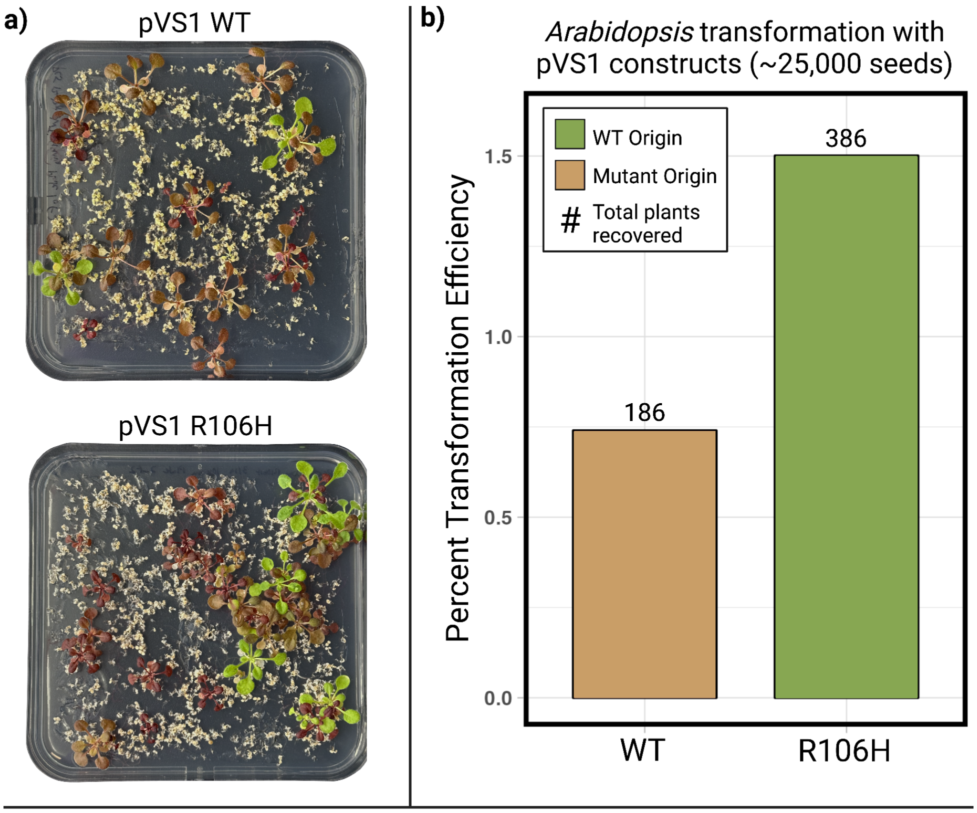

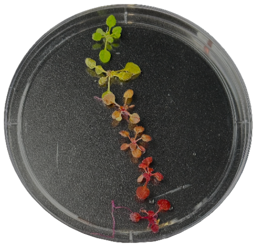

d)

Construct	pVS1_WT	pVS1_R106H
Seed (mg)	504	515
Seed Number	25200	25750
Plants Transformed	186	386
Percent Efficiency	0.74	1.50

Supplementary Figure 12: Validation of *A. thaliana* transformation improvement with the pVS1 R106H mutant using a *Ruby* construct. a) Representative plates of *A. thaliana* recovered on a MS-hygromycin medium for the pVS1_WT and pVS1_R106H constructs **b)** A bar graph depicting the transformation efficiency of both constructs when planting ~25,000 seeds. The total number of recovered plants is shown above each bar. **c)** Representative plants displaying a range of expression levels of the *Ruby* cassette, demonstrating recovered plants are transgenic. Phenotypically green plants grow on hygromycin and have a faint red tint in their roots and trichomes. **d)** A table recording the exact seed weights and corresponding seed number planted for each construct along with the total number of recovered plants and the transformation efficiency

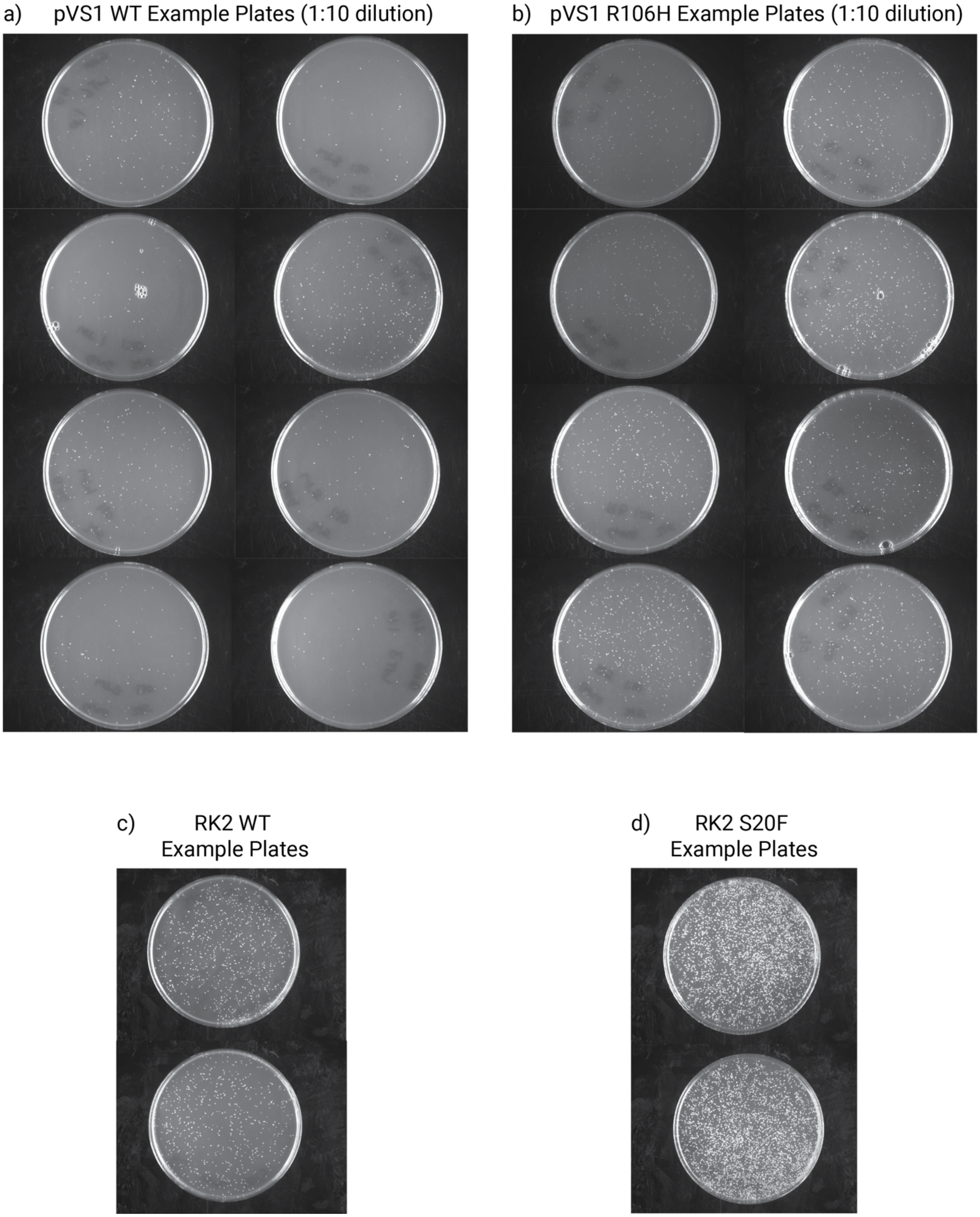

Supplementary Figure 13: Example *Rhodosporidium toruloides* plates

Example plates from transformations with pVS1 vectors in their WT (**a**) or R106H mutant form (**b**) are shown along with RK2 vectors in their WT (**c**) or S20F mutant form (**d**).
