# Supplementary material for: Binary vector copy number engineering improves Agrobacterium-mediated transformation
Source: Nat Biotechnol. 2024 Nov 4;43(10):1708–16. doi: 10.1038/s41587-024-02462-2 (PMC12147919; doi:10.1038/s41587-024-02462-2)
Supplement: Supplementary file 2 — Reporting Summary [file 41587_2024_2462_MOESM2_ESM.pdf]

nature portfolio

Corresponding author(s): Patrick M. Shih, Mitchell G. Thompson

Last updated by author(s): 07-30-2024

# Reporting Summary

Nature Portfolio wishes to improve the reproducibility of the work that we publish. This form provides structure for consistency and transparency in reporting. For further information on Nature Portfolio policies, see our Editorial Policies and the Editorial Policy Checklist.

## Statistics

For all statistical analyses, confirm that the following items are present in the figure legend, table legend, main text, or Methods section.

| n/a | Confirmed | |
|---|---|---|
| ☐ | ☒ | The exact sample size (*n*) for each experimental group/condition, given as a discrete number and unit of measurement |
| ☐ | ☒ | A statement on whether measurements were taken from distinct samples or whether the same sample was measured repeatedly |
| ☐ | ☒ | The statistical test(s) used AND whether they are one- or two-sided<br>*Only common tests should be described solely by name; describe more complex techniques in the Methods section.* |
| ☒ | ☐ | A description of all covariates tested |
| ☐ | ☒ | A description of any assumptions or corrections, such as tests of normality and adjustment for multiple comparisons |
| ☐ | ☒ | A full description of the statistical parameters including central tendency (e.g. means) or other basic estimates (e.g. regression coefficient) AND variation (e.g. standard deviation) or associated estimates of uncertainty (e.g. confidence intervals) |
| ☐ | ☒ | For null hypothesis testing, the test statistic (e.g. *F*, *t*, *r*) with confidence intervals, effect sizes, degrees of freedom and *P* value noted<br>*Give P values as exact values whenever suitable.* |
| ☒ | ☐ | For Bayesian analysis, information on the choice of priors and Markov chain Monte Carlo settings |
| ☒ | ☐ | For hierarchical and complex designs, identification of the appropriate level for tests and full reporting of outcomes |
| ☐ | ☒ | Estimates of effect sizes (e.g. Cohen's *d*, Pearson's *r*), indicating how they were calculated |

*Our web collection on statistics for biologists contains articles on many of the points above.*

## Software and code

Policy information about availability of computer code

| | |
|---|---|
| Data collection | No custom code was used to collect data |
| Data analysis | Quality control for the reads from the NGS runs was performed using FastQC v0.11.8 and MultiQC v1.10.1. Read trimming was performed using trimmomatic v0.39. Trimmed reads were aligned to their corresponding reference file using Bowtie v2.4.5. Mutations in sequencing reads were identified using pysamstats v1.1.2 and pysam v0.18.0. All code from this work is publicly available on GitHub (https://github.com/shih-lab/origin_story), and all sequencing reads are available on the NCBI SRA under BioProject accession PRJNA1031697. All plasmids generated in this paper were designed using Device Editor which is a part of the DIVA suite v6.1.2 and Open Vector Editor software v18.3.6 while all primers used for the construction of plasmids were designed using j5 software v3.4.0. |

For manuscripts utilizing custom algorithms or software that are central to the research but not yet described in published literature, software must be made available to editors and reviewers. We strongly encourage code deposition in a community repository (e.g. GitHub). See the Nature Portfolio guidelines for submitting code & software for further information.

# Data

Policy information about availability of data

All manuscripts must include a data availability statement. This statement should provide the following information, where applicable:

- Accession codes, unique identifiers, or web links for publicly available datasets
- A description of any restrictions on data availability
- For clinical datasets or third party data, please ensure that the statement adheres to our policy

All NGS data can be found on the NCBI SRA under BioProject accession PRJNA1031697 , the analysis can be found on our github https://github.com/shih-lab/origin_story, and all sequences from this work can be found at the following link https://public-registry.jbei.org/folders/847

# Research involving human participants, their data, or biological material

Policy information about studies with human participants or human data. See also policy information about sex, gender (identity/presentation), and sexual orientation and race, ethnicity and racism.

| | |
|---|---|
| Reporting on sex and gender | NA |
| Reporting on race, ethnicity, or other socially relevant groupings | NA |
| Population characteristics | NA |
| Recruitment | NA |
| Ethics oversight | NA |

Note that full information on the approval of the study protocol must also be provided in the manuscript.

# Field-specific reporting

Please select the one below that is the best fit for your research. If you are not sure, read the appropriate sections before making your selection.

☒ Life sciences ☐ Behavioural & social sciences ☐ Ecological, evolutionary & environmental sciences

For a reference copy of the document with all sections, see nature.com/documents/nr-reporting-summary-flat.pdf

# Life sciences study design

All studies must disclose on these points even when the disclosure is negative.

| | |
|---|---|
| Sample size | 6 tobacco plants were used for the infiltration experiments with data taken from 2 independent leaves with 4 leaf punches per leaf for a total of 48 independent data points per plant. Sample sizes were chosen based on known variation between leaves and plants in N. benthamiana and for statistical rigor to detect potential differences between samples, and this size exceeds the standards set by other leading studies in the field that use N. benthamiana as a screening tool (PMID: 35951698, 37348464, 37558215). Sample sizes for this study meet or exceed the standards in the field for transient tobacco assays. Sample sizes for Arabidopsis were set at the ~25,000 seed threshold for statistical rigor to be able to recover hundreds of transformation events to determine if differences were present, and this size was set to meet or exceed precedence of comparing transformation efficiency in the literature (e.g. PMID: 23581821). At least 15 plants were used per construct to allow for an assay of transformation efficiency over multiple individuals. 10 independent transformations were conducted for Rhodosporidium experiments for statistical robustness due to known variation in this transformation protocol and from previous literature (e.g. PMID: 32024522, https://doi.org/10.1039/C9GC00920E) |
| Data exclusions | No data was excluded |
| Replication | 6 plants per construct were used for all tobacco infiltration experiments. Experiments that determined stable transformation efficiency were repeated at least twice on separate dates with 2 floral dips conducted on each plant per replicate. A total of at least 15 independent plants were used per construct for the Arabidopsis experiments. 10 independent transformation replicates were conducted for the Rhodosporidium experiments. Data from replicates are reported are were successful. |
| Randomization | Plants were randomly selected for infiltration (N. benthamiana and Lactuca sativa) or floral dip (Arabidopsis thaliana). |
| Blinding | No blinding was used for the plant experiments due to the nature of characterizing a large number of ORI variants. As no information about potential ORI performance was known a priori, blinding was not utilized. |

# Reporting for specific materials, systems and methods

We require information from authors about some types of materials, experimental systems and methods used in many studies. Here, indicate whether each material, system or method listed is relevant to your study. If you are not sure if a list item applies to your research, read the appropriate section before selecting a response.

## Materials & experimental systems

| n/a | Involved in the study | |
|---|---|---|
| ☒ | ☐ | Antibodies |
| ☒ | ☐ | Eukaryotic cell lines |
| ☒ | ☐ | Palaeontology and archaeology |
| ☒ | ☐ | Animals and other organisms |
| ☒ | ☐ | Clinical data |
| ☒ | ☐ | Dual use research of concern |
| ☐ | ☒ | Plants |

## Methods

| n/a | Involved in the study | |
|---|---|---|
| ☒ | ☐ | ChIP-seq |
| ☒ | ☐ | Flow cytometry |
| ☒ | ☐ | MRI-based neuroimaging |

# Dual use research of concern

Policy information about dual use research of concern

## Hazards

Could the accidental, deliberate or reckless misuse of agents or technologies generated in the work, or the application of information presented in the manuscript, pose a threat to:

| No | Yes | |
|---|---|---|
| ☒ | ☐ | Public health |
| ☒ | ☐ | National security |
| ☒ | ☐ | Crops and/or livestock |
| ☒ | ☐ | Ecosystems |
| ☒ | ☐ | Any other significant area |

## Experiments of concern

Does the work involve any of these experiments of concern:

| No | Yes | |
|---|---|---|
| ☒ | ☐ | Demonstrate how to render a vaccine ineffective |
| ☒ | ☐ | Confer resistance to therapeutically useful antibiotics or antiviral agents |
| ☒ | ☐ | Enhance the virulence of a pathogen or render a nonpathogen virulent |
| ☒ | ☐ | Increase transmissibility of a pathogen |
| ☒ | ☐ | Alter the host range of a pathogen |
| ☒ | ☐ | Enable evasion of diagnostic/detection modalities |
| ☒ | ☐ | Enable the weaponization of a biological agent or toxin |
| ☒ | ☐ | Any other potentially harmful combination of experiments and agents |

## Plants

| | |
|---|---|
| Seed stocks | Col-0 seeds for Arabidopsis were obtained from the ABRC. |
| Novel plant genotypes | No novel plant genotypes were used in this study. Only WT plants were assayed for all experiments. |
| Authentication | NA |
